# Electrochemical Capacitance of CNF–Ti_3_C_2_T_x_ MXene-Based Composite Cryogels in Different Electrolyte Solutions for an Eco-Friendly Supercapacitor

**DOI:** 10.3390/gels11040265

**Published:** 2025-04-03

**Authors:** Vanja Kokol, Subramanian Lakshmanan, Vera Vivod

**Affiliations:** Faculty of Mechanical Engineering, University of Maribor, Smetanova ul. 17, 2000 Maribor, Slovenia; subramanian.lakshma1@student.um.si (S.L.);

**Keywords:** cellulose nanofibrils, Ti_3_C_2_T_x_ MXene, freeze-casting, aqueous electrolytes, physico-chemical properties, electric double layer, pseudocapacitance

## Abstract

Cellulose nanofibrils (CNFs) are promising materials for flexible and green supercapacitor electrodes, while Ti_3_C_2_T_x_ MXene exhibits high specific capacitance. However, the diffusion limitation of ions and chemical instability in the generally used highly basic (KOH, MXene oxidation) or acidic (H_2_SO_4_, CNF degradation) electrolytes limits their performance and durability. Herein, freestanding CNF/MXene cryogel membranes were prepared by deep freeze-casting (at −50 and −80 °C), using different weight percentages of components (10, 50, 90), and evaluated for their structural and physico-chemical stability in other less aggressive aqueous electrolyte solutions (Na_2_/Mg/Mn/K_2_-SO_4_, Na_2_CO_3_), to examine the influence of the ions transport on their pseudocapacitive properties. While the membrane prepared with 50 wt% (2.5 mg/cm^2^) of MXene loading at −80 °C shrank in a basic Na_2_CO_3_ electrolyte, the capacitance was performed via the forming of an electroactive layer on its interface, giving it high stability (90% after 3 days of cycling) but lower capacitance (8 F/g at 2 mV/s) than in H_2_SO_4_ (25 F/g). On the contrary, slightly acidic electrolytes extended the cations’ transport path due to excessive but still size-limited diffusion of the hydrated ions (SO_4_^2−^ > Na^+^ > Mn^2+^ > Mg^2+^) during membrane swelling, which blocked it, reducing the electroactive surface area and lowering conductivities (<3 F/g).

## 1. Introduction

Rechargeable electrochemical supercapacitors (SCs) with good flexibility have recently attracted considerable interest as energy storage devices in wearable and portable electronics [1], as they can provide sufficiently high power density with fast charging and discharging processes, along with long lifetime and stability. Depending on the type of energy storage mechanisms, SCs are categorised into electric double-layer capacitors (EDLCs, being usually activated carbon), and [2] pseudocapacitors (mostly conducting polymers and metal oxides or hydroxides) [3]. In such devices, two metal electrode plates are separated by a thin and porous insulator (made from carbon, paper, or plastic) soaked in an electrolyte. For EDLCs, electrostatic charge is accumulated at the electrode/electrolyte interface, while for the pseudocapacitor the active materials undergo a Faradaic redox reaction by a fast and reversible electron charge transfer between the electrode and electrolyte. This comes from the de-solvated and near-surface adsorbed electrolyte ions that migrate toward the electrode with the opposite polarity when a charge is applied [4]. The capacitance is thus influenced greatly by the electrode’s surface area and its accessibility to electrolyte ions. The introduction of an electrochemically inert binder (such as nanocellulose) to improve device durability and flexibility may thus affect electrode processing, stability, and irreversible capacity losses significantly [5] due to the kinetically limited ion diffusion into the bulk phase of the electrode.

Nanocellulose (NC), a sustainable, lightweight and environmentally friendly nanomaterial, is gaining interest for the production of green, inexpensive and recyclable electronic devices with potential applications in wearable sensors, healthcare monitoring and artificial intelligence [6]. Its attractive mechanical (tensile strength of ~14 GPa, Young’s modulus of ~100 GPa), physical–chemical (density of ~1.6 g/cm^3^, specific surface area of 200–300 m^2^/g, thermal expansion coefficient of ~1 ppm 1/K, high thermal stability) and dielectric properties [7,8] make NC an ideal building block for flexible functional materials, which are beneficial for energy conversion and conservation applications, including electrochemical energy storage [6,9,10]. The possibilities of using NC in the fabrication of electrodes, current collectors and separators for SC and batteries [11], and thereby the manufacturing of a complete all-cellulose-based electrochemical energy storage device have thus been attracting a lot of attention recently [6,12]. However, the energy storage properties of NC-based electrodes depend mainly on the homogeneous dispersion of the electronically conductive filler, its surface area, and interactions within the NC acting as a binder. Therefore, several fabrication strategies and protocols have been studied to improve ion and electron transport for their best performance (high enough areal/volumetric capacities/capacitances). The latter requires proper selections of both components, as well as optimised porosity to ensure high mass loading, an efficient charge–discharge rate and good cyclic performance; inadequate porosity either limits mass transport, thereby reducing power, or energy density (due to excessive electrolyte accumulation in the pores of the electrode, which blocks them, lowering the conductivities, and reducing the active surface area). For improved capacity (electrolyte uptake, ionic conductivity), as well as cyclic stability (mechanical stress and deformations) and performance (charge storage) of such composites, research has accelerated into NC-based aerogels and scaffold/membrane structures (prepared with different techniques, such as vacuum filtration, solvent casting, or solvent exchange) with integrated (immobilised into it or thin-layer coated onto it) carbon nanotubes [13], reduced graphene oxide (460 F/g; [14]) and graphene (120 F/g, [15,16]), metal particles (Ag) and conductive polymers (polypyrole, 320 F/g; [17]; Ag/polyaniline [18]). Such electrodes can exhibit excellent cycling performance (85–99% capacitance retained over 1000 cycles, Table S1), as well as exceptional rate capabilities when normalised with the integrated electroactive mass [19]. However, the chemical stability of cellulose in such a commonly used highly acidic (H_2_SO_4_) electrolyte solution could be problematic (degradation), while also reducing cations’ transport due to their interactions with the negatively charged functional groups on the NC’s surface under such conditions.

Titanium carbide, by far the most studied and well-understood type of MXene with the general formula Ti_3_C_2_T_x_, where T*_x_* denotes terminated hydrophilic surface functional groups (such as =O, –OH, and –F, generated during air oxidation and chemical preparation), are a new class of 2D nano-layered materials [20] with high surface area and fast monovalent cation (H^+^, Li^+^, Na^+^, K^+^) diffusion, beneficial for reversible electrochemical (surface redox) reactions with fast charge/discharge rates. This gives them high semiconductive or metallic electrical conductivity (even up to a few 1000 S/cm), [21], as well as excellent ion-embedded pseudocapacitive charge-storage capability [1]. This also distinguishes them from other 2D nanomaterials, above all graphene [22], and makes them promising candidates for applications in energy storage devices [23], such as SC electrodes [24] and rechargeable batteries [21], conductive films [25] and sensors [26]. Pristine Ti_3_C_2_T_x_ MXene can also be shaped into an adhesive-free anode with high specific (92 F/g in 3M KOH and 75 F/g in 3M Na_2_SO_4_ electrolytes at 2 A/g current density [27]) and volumetric (>300 F/cm^3^ at 2 mV/s in H_2_SO_4_ and Li_2_SO_4_) capacitances, which indicates that the alkaline electrolyte might also be a promising electrode when compared to acidic electrodes for SC applications. Recent studies suggest that even larger/bigger cations can insert and extract into the interlayer space of their 2D structure unboundedly [21,24]. Massive storage power was thus demonstrated with intercalated polyvalent cations, such as Ca^2+^ [28], Mg^2+^ [29,30], and Al^3+^ [31] ions. Several other factors also affect the electrolyte ions’ accessibility to the active sites by disrupting/hindering the electron pathways (transport/flow/movement), and thus reducing the overall capacitance performance of MXene, such as the alteration and configuration of the surface terminal groups, intercalated species (ions or molecules between their layers), defects and disorders, layer stacking (weaker interlayer interactions), the formation of insulating surface oxides, and a tendency to self-assemble or aggregate due to strong van der Waals forces and hydrogen/H-bonding between the adjacent MXene nanolayers. To address these issues, many strategies have been proposed, including the intercalation and terminal groups’ removal [32], the introduction of interlayer spacers [33], porous macroscopic structures prepared by, e.g., foaming [34] or ice-templating/freeze-drying [19,35,36] from a highly concentrated MXene dispersion. NC with a large aspect ratio (a width of up to a few 10 nm and length of up to 10 μm) and abundant hydroxyl/–OH and other oxygen polar groups, has also been used as a spacer to increase the distance between the Ti_3_C_2_T_x_ interlayer flakes, thereby providing more H-bonding sites and stronger entanglement during their mixing, resulting in enhanced mechanical properties of such composites, as well as creating more ion-accessible surface areas on the Ti_3_C_2_T_x_ for faster ion transport. For example (Table S1), the use of bacterial cellulose in stretchable and flexible all-solid-state SCs [6] showed the provision of tunnels between the Ti_3_C_2_T*_z_ MXene* flakes upon freeze-drying, which improved the capacitance for such a composite (416 F/g, 2084 mF/cm^2^) with high MXene loading (~5 mg/cm^2^). The use of Ti_3_C_2_T_x_ MXene with carboxymethylated CNFs [37] and sulphated CNFs [38] thereby facilitates high-performing SCs (298 and 191–220 F/g), with good flexibility at even up to 20 wt% of CNF loading. Simple structural engineering strategies obtained by vacuum- or freeze-drying were proposed to control such a composite interlayer structure of composite films, also showing excellent cycle performance (100% capacitance retention rate after 5000 cycles) [38]. Microfibrillated cellulose film, surface coated with Ti_3_C_2_T_x_ MXene, also endowed accessibility of the electrolytes to the Ti_3_C_2_T_x_ surface with fast ion transport and delivered high specific capacitance (451 F/g, [39]. The presence of reduced graphene oxide [40], porous carbon [2] or liquid metal [41], in combination with CNFs prepared by the vacuum-filtration or freeze-drying method additionally increases the interlayer distance between the Ti_3_C_2_T_x_ flakes, thereby demonstrating high areal capacitance (0.14–0.87 F/cm^2^) with ~50% retention.

However, the electrolyte ions’ (both cations and anions) migration and interaction with the (potentially ionised) surface functional groups on NC also affect the process of charging and discharging, and the overall capacities of such a hybrid composite. Pseudocapacitance is generated if the electrolyte cations are small enough to pass through such a composite structure without interacting with the negatively charged NC surface and thus move to and through the MXene layers. In contrast, larger ions that cannot penetrate through both the NC barrier and Ti_3_C_2_T_x_ layers (or interacting with it ionically) create an electroactive layer by electrochemical adsorption. The design of such electrodes with a high and easily accessible electroactive mass (>10 mg/cm^2^) and relatively low amount of binder (5–10 wt%) is, therefore, still a major challenge [5]. A porous cellulose-based electrode with a thin layer of deposited electroactive material [39] can provide the large surface area that is required, and, at the same time, enables rapid mass transport by utilising the entire volume of such an electrode.

In this study, the one-directional freeze-casting method was thus applied to prepare such a porous structured electrode, using cellulose nanofibrils (CNFs) and Ti_3_C_2_T_x_ MXene, that is formed by electrostatic self-assembly interfacial interactions between the water-suspended components during kinetically different water crystallisation (which was performed at −50 °C and −80 °C). The effect of the CNF and MXene weight percentage ratio (90:10, 50:50, 10:90) on their morphology, porosity and adsorption isotherms/capacity of different aqueous electrolytes has been studied to gain insight on their long-term stability (mechanical and chemical), as well as electrochemical performances. Due to the different solubility of electrolytes at room temperature [3], 1–0.5M concentrated electrolyte solutions were used, all providing high conductivity and the ability to operate in a wide potential window (up to about 1.6 V). Along with the most commonly used, although aggressive, acidic (H_2_SO_4_ ~338 mS) and alkaline (KOH ~159 mS) solutions, less toxic, corrosive, non-flammable, cost-efficient and pH slightly acidic (pH~4.8–6.0)/basic (pH~11) aqueous electrolytes (i.e., Na_2_SO_4_ ~79 mS, MgSO_4_ ~42 mS, MnSO_4_ ~37 mS, K_2_SO_4_ ~69 mS, and Na_2_CO_3_ ~70 mS) were studied as alternatives [5,42]. Thereby, the influence was examined by using other cations (Mg^2+^, Mn^2+^) or anions (CO_3_^−2^), instead of H^+^ or K^+^ and OH^−^ or SO_4_^2−^, on the physico-chemical and electrochemical properties of such prepared membranes. The long-term CV plots were performed in addition as a function of scan rates to gain insight into specific capacitance changes and membrane stability.

## 2. Results and Discussion

### 2.1. Preparation and Characterisation of Ti_3_C_2_T_x_ MXene

In the Ti_3_AlC_2_ MAX phases, where the Ti-C bond is stronger than the Ti-Al bond, the Al layer can be removed by suitable etching reagents, producing multilayered stacked Ti_3_C_2_T_x_ MXenes, which are then stabilised through H-bonds and van der Waals forces between the 2D nano-thin flakes of Ti_3_C_2_T_x_ [43]. Multilayered MXenes can be delaminated further and intercalated to thinner single/few-layered Ti_3_C_2_T_x_ MXenes to increase their surface area and produce electrochemically more attractive materials.

**Figure 1:**
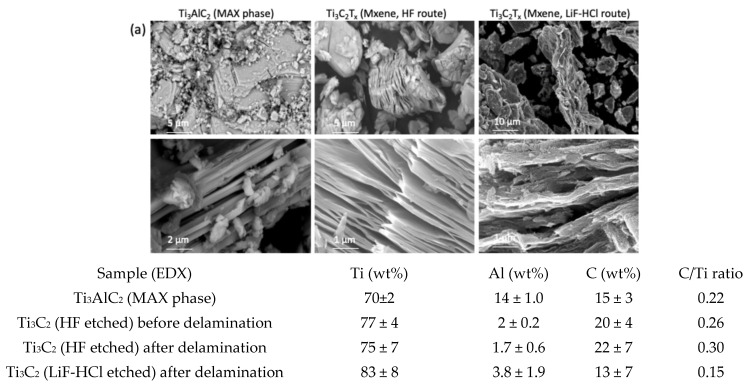

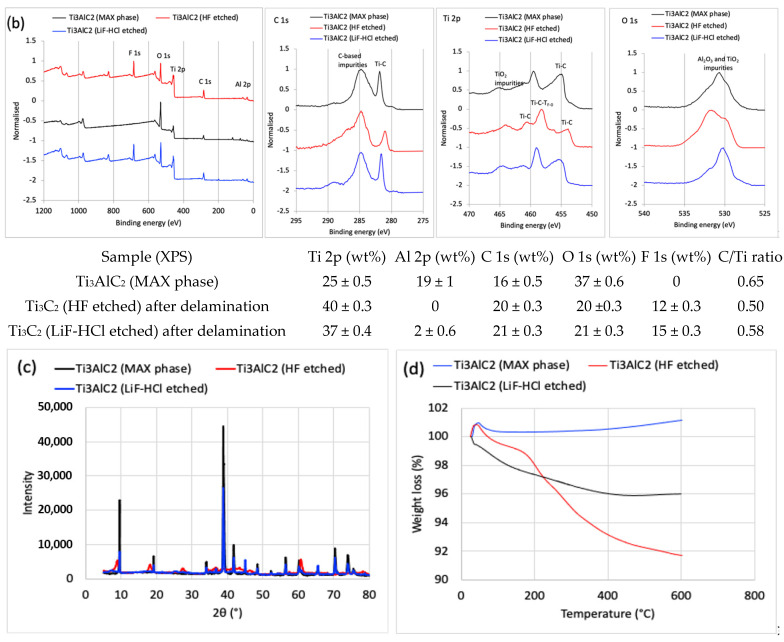
Morphological and structural characterisation of differently prepared Ti_3_C_2_T_x_ MXenes from Ti_3_AlC_2_ (MAX phase) by (**a**) SEM imaging supported with EDX elementary analysis (Table), (**b**) XPS spectra with corresponding bending energy values (Table) associated with specific elements, (**c**) XRD, and (**d**) TGA analysis.

The preparation of Ti_3_C_2_T_x_ MXene and their delamination efficacy from the Ti_3_AlC_2_ MAX phase powder was studied by SEM imaging connected to EDX, as well as XPS and XRD spectroscopies, and TGA analysis. The SEM images (Figure 1a) and EDX spectra (elemental composition in the inserted Table), performed within the same view field, showed that most of the Al in the Ti_3_AlC_2_ MAX phase was removed after the HF etching, resulting in a typical accordion-like morphology of the multi-layered (up to ca. 20) and few 10-nm thick Ti_3_C_2_T_x_ MXene sheets, with a distance between successive layers of around 100–200 nm after delamination and the MXene of several microns in lateral sizes. The total weight percent of Al was reduced from ~27% in the MAX phase to ~1.9% after etching with HF, and reduced further to ~1.7% after the delamination step, while Ti increased by ~24% and the carbon remained constant, confirming the almost complete elimination of the Ti-Al bonds and optionally present C-Al bonds in the so obtained Ti_3_C_2_T_x_ MXene. XPS (Figure 1b) analysis also confirmed the presence of terminated (T_x_) groups (such as =O, –OH, and –F) on the surfaces of the MXene layers by the peaks of O 1s at ~529 eV (LiF-HCl etched) and ~531 eV (HF etched), and the peak of F 1s at ~684 eV, respectively [34]. The Ti–C components, whose binding energy positions depend on the local bonding of the terminal –F and oxygen (=O, –OH) species, are also confirmed by the Ti 2p spectra. The Ti_3_C_2_T_x_ MXene obtained instead had the expected C/Ti atomic concentration ratio of ~0.6 (3:2). The presence of C-based impurities, such as hydrocarbons (CH), alcohol (C–OH), and carboxyl (COO) components, can also be confirmed by the C 1s spectra [20]. From a relatively high percentage of oxygen components in O 1s and shoulder peak at ~463 eV for the Ti 2p spectra, some of them also have to be in the form of TiO_2_ (and possible Al_2_O_3_), probably caused by exposure of samples to the laboratory atmosphere, strong ultrasonication and centrifugation during the preparation phase; it could be expected that the oxidised Ti and Al were also present on the interface of the layered MXene structure (where they interacted with the conductive Ti–C), as estimated from the EDX analysis. Enhanced oxidation might also be the reason for the higher adhesion between the MXene layers, inhibiting their delamination. No Li, but a small Cl residual in the MXene exfoliated by LiF-HCl was detected, indicating its incomplete removal during washing. The XRD patterns (Figure 1c) additionally confirmed the selective removal of Al and the formation of a Ti_3_C_2_T_x_ crystal structure, characterised by the presence of characteristic diffraction peaks at 2θ at ~8.7°, ~18°, ~29°, and ~36°, ~45° and ~61°, corresponding the to (002), (004), (006), and (008), (010) and (110) planes of the Ti_3_C_2_T_x_ MXene, respectively [20]. A noticeable downshift in the (002) diffraction of Ti_3_AlC_2_ from ~9.5° to ~8.7° in the Ti_3_C_2_T_x_ also indicates the introduction of surface terminal groups in the structural composition of the Ti_3_C_2_T_x_, which corresponds to an increase in the spacing between the 2D layer stacks and a decline in their thickness, as is evident from the SEM images. The large spacing after drying could also be due to the remaining water molecules in between the MXene layers, which was confirmed by the TGA thermograms (Figure 1d), showing a multi-stage reduction in weight by increasing temperature. The first two stages are due to the loss of physically and chemically (via –OH groups on Ti_3_C_2_ surface) adsorbed water, and the third is related to the loss of adsorbed –F (also via –OH groups); the latter can be supported by the XPS analysis, according to which the F/O atomic concentration ratio on the MXene surface was ~0.6 for the etched HT and ~0.49 for the LiF-HCl etched MXene.

### 2.2. Structural and Physico-Chemical Properties of Composite Membranes

**Figure 2:**
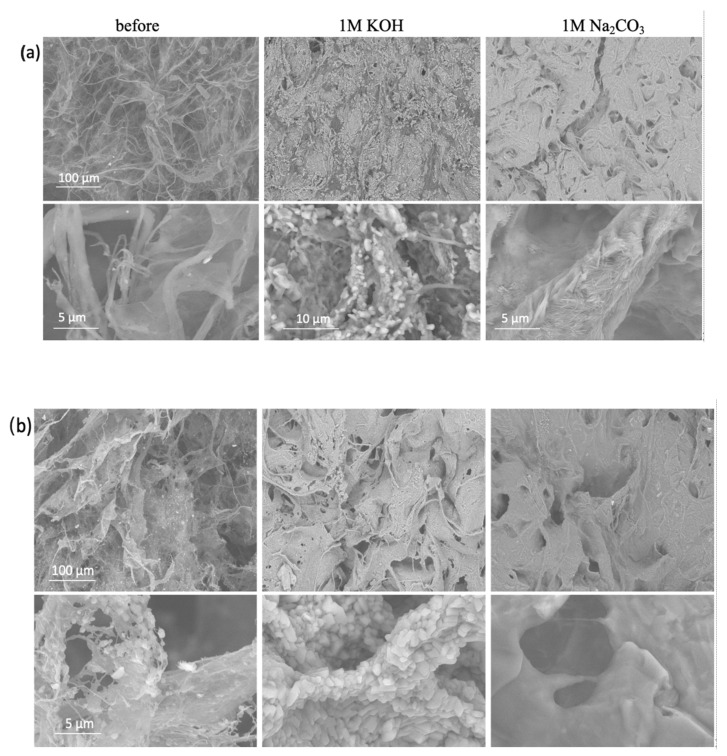

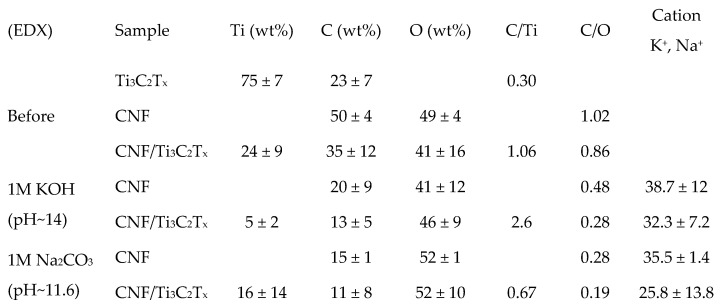
SEM images with EDX elementary analysis (inserted Table) of a CNF-based membrane outer surface prepared (**a**) without and (**b**) with 50 wt% Ti_3_C_2_T_x_ MXene (HT-etched) at −50 °C, before and after 24-h immersion in selected (alkaline) electrolyte solutions.

The surface and bulk/volumetric densities and total porosity of the selected composite membranes, prepared at both freezing temperatures (−50 °C and −80 °C) and containing different concentrations of HF-etched Ti_3_C_2_T_x_ MXene, were evaluated by gravimetric analysis (Table 1) to characterise their morphological structure. The average volumetric density was decreased from ~289% for the sample prepared with 50 wt% of MXene at −50 °C to ~229% for the same prepared at −80 °C, at similar (99.8–99.3%) porosities. This effect was even more significant in membranes containing 90 wt% MXene. The results are supported by the SEM images of representative samples, presented in Figure 2, that indicated a fully open web-like net microstructure, with disorderly arranged bundles and single cellulose nanofibrils in the case of membranes prepared with pure CNF. On the other hand, a denser microstructure was observed for the sample with the presence of MXene, also indicating an orientation of CNFs along the Mxene sheets and their high adhesion. Accordingly, an increase in the MXene content resulted in an even denser micro-porous structure, being more pronounced for samples prepared at lower temperatures (−80 °C), having also a slightly lower porosity than those prepared at −50 °C. The process is related to the different freezing kinetics of the water molecules (i.e., water crystallisation growth and structuring), and, thus, the creation of differently sized and distributed ice crystals around the CNFs and MXenes, which became smaller and more ordered at lower temperature, therefore also influencing their assembling and rearrangement dynamics.

**Figure 3:**
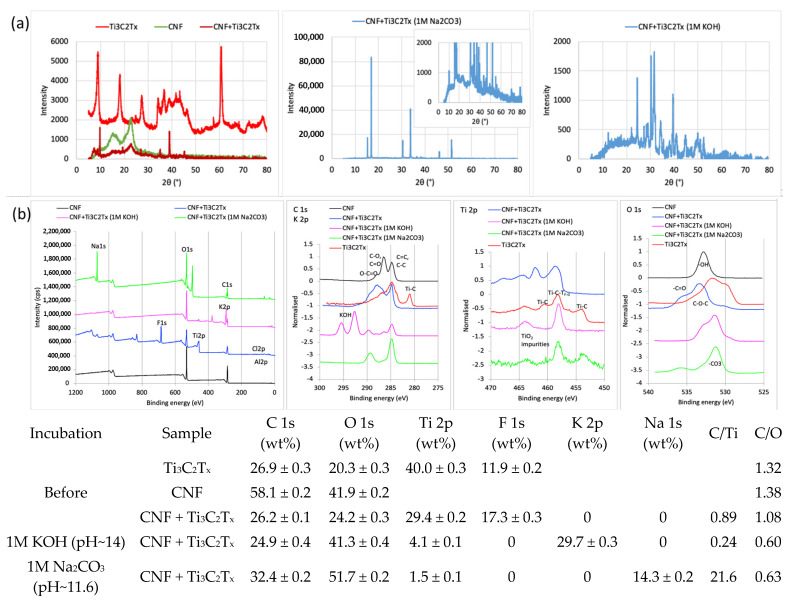
(**a**) XRD and (**b**) XPS spectra of Ti_3_C_2_T_x_ MXene, CNF and CNF/MXene composite membranes prepared with 50 wt% Ti_3_C_2_T_x_ MXene (HT-etched) at −80 °C, before and after 24-h immersion in selected (alkaline) electrolyte solutions.

The elemental compositions obtained by EDX elementary analysis (and some of the elements’ ratios, Figure 2, inserted Table) of the representative CNF/MXene hybrid membranes also confirmed the integration of Ti_3_C_2_T_x_ MXene in the fibrillated cellulose network by the appearance of Ti, and significant reduction of C and O content and its C/O ratio from ~1 to ~0.86. The XRD spectra of such a hybrid membrane (Figure 3a) additionally revealed a slight shifting of the main characteristic diffraction peak of MXene to a higher angle (from ~8.7° to ~9.8°), and a significant drop in the intensity of the others after the introduction of CNF. At the same time, the amorphous cellulose peaks at 2θ of ~16° and ~18° (attributed to the (1–10) and (110) planes), was reduced more strongly than the peak for crystalline cellulose at ~22.8° (corresponding to the (200) plane, of cellulose I_β_); [44] indicated the covering/interactions of the Ti_3_C_2_T_x_ MXene surface by CNFs [43], and possible intercalation between the Ti_3_C_2_T_x_ interlayers by smaller CNFs fractions, that can also prevent the restacking of delaminated MXene flakes [45], and, consequently, enhanced accessibility of the electrolyte and accommodation of more ions. The chemical bonding states between the components on the outside of the hybrid membranes surface was confirmed additionally by FTIR and XPS spectroscopy. The IR spectrum of the CNF membrane (Figure S1 showed characteristic transmittance bands for cellulose, with a broad peak at ~3337 cm^1^ (assigned to –OH stretching), ~2900 cm^1^ (–CH_2_ stretching), ~1410–1420 cm^−1^ (–CH_2_ bending), ~1360–1310 cm^−1^ and ~1150–1110 cm^−1^ (–CH stretching and bending), ~1030 cm^−1^ (C-O-C stretching and −OH bending), ~890 cm^−1^ (cellulosic *β*-glycosidic linkages), and ~660 cm^−1^ (−OH bending); a small shoulder at ~1600 cm^−1^, attributed to the stretching vibrations of C=O, also indicated the presence of −COOH groups. The spectra of the Ti_3_C_2_T_x_ MXene powder had small shoulders at ~3400 cm^−1^, ~1000 cm^−1^, and more intensive ones at ~600–500 cm^−1^, corresponding to the stretching vibrations of the –OH, C–F, and Ti–O bonds, respectively [46]. All the characteristic peaks for the composite membranes were shifting slightly and obviously decreased as compared to the solvent-casted films and CNFs, indicating the formation of H-bonding between the −OH, −O and C=O atoms in the CNF with the surface groups of Ti_3_C_2_T_x_ (=O, –OH, and –F). The presence of a small shoulder for the −OH groups (at ~3334 cm^−1^ and ~1030 cm^−1^) confirmed their availability for interaction with the electrolyte cations; no differences were observed between the upper and bottom sides of the samples. The XPS spectra (Figure 3b) reconfirmed the presence of Ti, C, O, and F for the composite membrane, while the deconvoluted Ti 2p spectra exhibited an additional peak for Ti-O at ~461.8 eV as compared to the Ti_3_C_2_T_x_ sample after mixing with CNF, additionally confirming their interaction with the surface-decorated −OH and rare −COOH groups of CNF [2]. The deconvoluted C 1s spectra contained a typical peak at ~286.2 eV (Ti–C) in the composite membrane, where the C/O-enriched structure of pure CNF was well-maintained by showing a broadened peak at ~287.2 eV corresponding to the C=C/C–C in aromatic rings, C–O and C=O, and a small shoulder at ~289 eV of O–C=O. On the other hand, a shifting of a broadened O peak to higher binding energy (~535 eV and ~533 eV) in the O 1s spectra indicates the formation of new—via O and C atoms—linked structures, although the broadened peak at ~532.6 eV can also be assigned to the –OH of the bound water. The results of the XPS were in line with the elemental mapping obtained by EDX spectrometry, and both results confirmed the structural integrity of Ti_3_C_2_T_x_ MXene within the CNF matrix and their intercalations. Besides the H-bonding and stronger van der Waals forces, electrostatic repulsions might also be present, influencing their morphological structure.

### 2.3. Structural and Physico-Chemical Stability of Composite Membranes in Different Electrolyte Solutions

**Figure 4:**
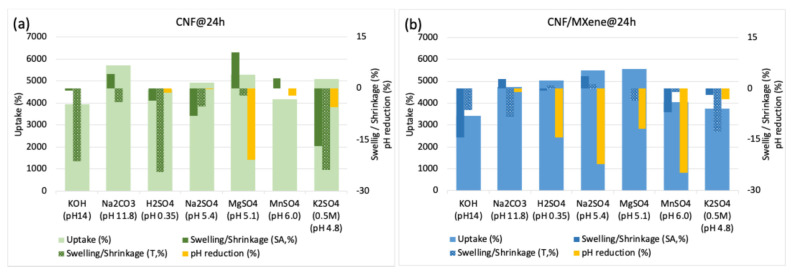
Characterisation of (**a**) Pure CNF, and (**b**) CNF/Ti_3_C_2_T_x_ MXene membranes prepared with 50 wt% MXene (HF-etched) at −80 °C freeze-casting temperature, after 24 h of immersion in different 1/0.5M electrolyte solutions: The uptake of electrolyte solutions, The swelling/shrinkage percentage based on surface area (SA) and thickness (T) change, and The reduction of solutions pH after 7 days of membrane incubation.

In addition to the conductivity and hydrodynamic size of the electrolyte ions, the wetting behaviour of the membrane in the electrolyte solution also facilitated cation diffusion and thus affected their electrochemical properties. The membranes prepared without and with (HT-etched) MXene at both freezing temperatures were thus immersed into different electrolyte solutions to evaluate their swelling, electrolyte uptake, and interaction with CNFs/MXene, as well as their stability. The membranes prepared with the highest (90 wt%) MXene loading disintegrated after 24 h of incubation in all the aqueous electrolyte solutions (Figure S2), so they were unsuitable for further analysis. The 1M H_2_SO_4_ solution was also proved to be a mechanically unsuitable electrolyte for all the CNF-based membranes due to cellulose depolymerisation in such a highly acidic medium (pH~0.35) [35]; this was confirmed by significant shrinkage (Figure S3) of the pure CNF sample after 24 h of incubation, and its complete degradation after one week, resulting in glucose and sulphone-acid-decorated MXene particles, as observed from the photo and SEM image presented in Figure S3 (the spectroscopic elemental analysis of this sample could thus not be performed). On the other hand, the pure CNF membranes behaved differently in the other types of electrolytes (Figure 4a). While they floated in Na_2_SO_4_ and MnSO_4_ solutions of pH~5.2 and got slightly colourised/stained in MnSO_4_ (pH~6.0) due to Mn^2+^ adsorption, they did not show any optical difference generally in any of these electrolytes, including MgSO_4_ and K_2_SO_4_, even after 24 h of incubation (Figure S1). The solution (and, consequently, ion) uptake (Figure 4a) was also significantly lower for KOH (pH~14) and MnSO_4_ (pH~6.0) (~4000%) as compared to the other electrolytes (~4500–5700%). Still, it showed a significant shrinkage (areal and volumetric, ~16 and ~24%) in K_2_SO_4_ (pH~4.8) and slight shrinkage (~8 and ~5%) in Na_2_SO_4_ (pH~5.4), and in electrolytes with monovalent cations, a swelling (~10%) in MgSO_4_ (pH~5.1), while it did not affecting the dimensional change in MnSO_4_ (pH ~6.0). While the swelling in MgSO_4_ was related to high adsorption of hygroscopic Mg salts, resulting in a ~21% reduction of pH (from ~5.1 to ~4.0, the uptake of the solution (~5700%) without dimensional and pH change was the highest and most stable in Na_2_CO_3_ (pH~11.8). Such a behaviour might be due to different interactions of electrolyte cations with anionic (abundant -OH and rare -COOH) surface groups on the CNFs, where, besides hydrogen bonding and electrostatic attractive interactions (the latter is highly dependent on the electrolyte pH that affect both the ionization of functional groups on CNF and the dissociation degree of ions), the internal bonding of the fibril-to-fibril network could thus also be present in the case of electrolytes with divalent cations (above all, hydrodynamically larger Mg^2+^) that influenced the membrane’s physical properties. In highly basic electrolytes, the diffusion of cations is thus significantly reduced due their predominant electrostatic interactions with CNF functional groups already on the outer surface of the membrane walls, and the formation of salt aggregates or protective layers (Figure 2a) with their anions, which actually affects the high value of electrolyte uptake.

The membranes prepared with 50 wt% MXene loading (Figure 4b; for 10 wt% MXene loading the results are presented in Figure S4), followed a similar trend, but showed generally lower (by between 5 and 30%) electrolyte uptake, and, consequently, much lower shrinkage (areal and volumetric, ~0–14%) for samples prepared at −80 °C than those prepared at −50 °C (showing more intensive shrinkage in thickness, ~16–43%), which is related to the slightly higher density of those membranes, also influencing the accessibility and diffusivity of the ions (and, hence, shorter diffusion distances within the electroactive MXene). As already discussed above, besides pH (influencing the ionic form of –OH and rare –COOH groups on the CNF and ionization stage of electrolyte), such an effect could also be related to the size and form of the hydrated ions, and their ionic conductivity and mobility in an aqueous environment. Among them, the hydrodynamically smallest H^+^ ions (with a radius of 0.28 nm) had the highest conductivity (~350 Scm^2^/mol) and ionic mobility (~36.23 10^−8^ m^2^/sV), followed by OH^−^ (0.3 nm, ~198 Scm^2^/mol, ~20.6 10^−8^ m^2^/sV), K^+^ (0.331 nm, ~73.5 Scm^2^/mol, ~7.62 10^−8^ m^2^/sV), SO_4_^2−^ (0.379 nm, ~160 Scm^2^/mol, ~8.3 10^−8^ m^2^/sV), Na^+^ (0.358 nm, ~50.11 Scm^2^/mol, ~5.19 10^−8^ m^2^/sV), and Mg^2+^ (0.427 nm, ~106 Scm^2^/mol) [3,47]. A larger swelling and, thus, capacitance, can, therefore, be expected for electrolyte ions with different sizes, e.g., large anions and much smaller cations, where the valence of the ions and electrolyte pH also determines their interactions at the membrane–electrolyte interfaces and its stability [48]. For slightly acidic sulphated electrolytes, the sensitivity of cellulose -OH and rare -COOH groups (covering the MXenes) are relatively higher for hydrodynamically larger and slower divalent cations (Mg^2+^) than for the monovalent cations (as K^+^ and Na^+^) that can interact with hydrogen bonding, but also diffuse easier and faster to the MXenes’ surface where weakly adsorbed on their −F/−O/=O terminal groups (as confirmed by the XPS analysis presented in Figure 3 where rare Ti atoms and no F could be detected anymore on its surface) and, by this, also influence on ion selectivity. A higher pH reductio in the case of all sulphate electrolytes (Na_2_SO_4,_ MgSO_4_, MnSO_4_, KSO_4_), except H_2_SO_4,_ can thus be related to a much higher diffusion ability of the monovalent cations (Na^+^ > Mn^2+^ > Mg^2+^) and their interaction with available and rare ionically charged (at such a slightly acidic pH 4.8–5.6) functional groups on CNF, and their further diffusion to the MXene interface, or conjugation with SO_4_^2−^ during swelling and ion transfer. On the contrary, the diffusion of Na^+^ cations in highly basic Na_2_CO_3_ (pH~11.8) is more limited on the membrane outer surface due their predominant interactions with fully ionized functional surface groups on CNFs, and therefore also more prone to the formation of an electroactive layer on the membrane interface with larger CO_3_^−2^ anions, as is clearly evident from Figure 2b. The pronounced shrinkage across the thickness of samples with a more open structure prepared at −50 °C in all sulphate electrolytes, independent of the amount of MXen added (Figure S4b), thus causes stacking of diffused ions and limits the mass transfer during incubation, which actually affects the high value of electrolyte uptake, membrane density and pH reduction. In contrast, other slightly acidic electrolytes with divalent cations enable a higher rate of ion diffusion into the membrane structure.

**Figure 5:**
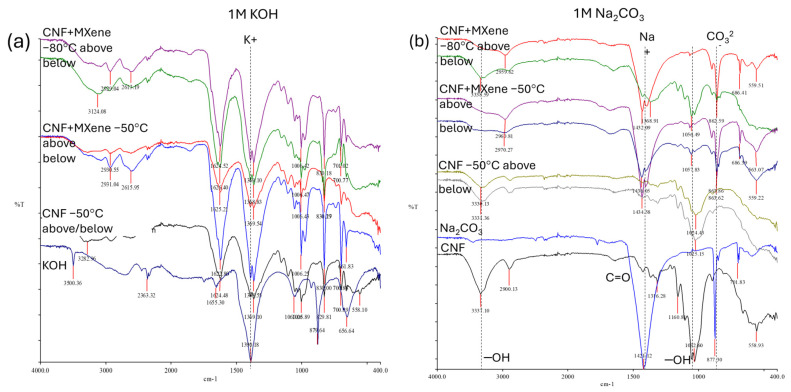

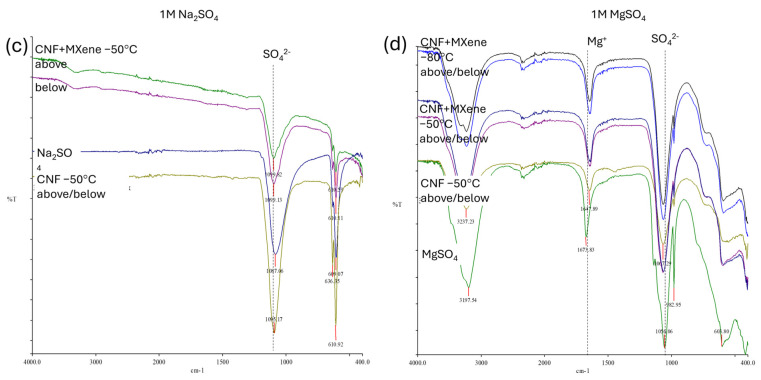
FTIR spectra of CNF/Ti_3_C_2_T_x_ MXene (HF-etched) membranes prepared at different freeze-casting temperatures (−50 °C vs. −80 °C) (**a,b**) after a few (2 or 4, respectively) days of CV, (**c,d**) or 7 days of immersion in different 1/0.5M electrolyte solutions, performed on both sides of the samples (the upper/air-exposed and the bottom/freezing-plate exposed side during freezing).

Such an interaction of ions with the CNF surface can also be confirmed by FTIR spectroscopy. As seen from the spectra in Figure S4c, all the characteristic peaks for CNFs were found in the IR spectra of the pure CNF membranes (prepared at −50 °C) after 7 days of incubation in different electrolyte salts, but with different intensities, while the new peaks—associated with the crystallite salts—appeared, indicating different associations of electrolyte ions with the CNFs’ surface. The bands assigned to the −OH surface groups (above all, those at ~3337 cm^−1^ and ~1030 cm^−1^) in the membranes after incubation were broadened, weakened, and/or shifted slightly to a lower (~3230–3282 cm^−1^) or higher (~1070–1095 cm^−1^) wavelength, depending on the electrolyte solution used, and, consequently, H-bonding between the anionic −OH and −O, as well as rare –COOH groups in the CNF with deposited electrolyte cation and the formation of crystal structures after its conjugation with anion on the anchoring sites on the CNF surfaces. In general, two new bands appeared in the IR spectrum of the CNF samples after incubation in sulphate salts, at ~1059–1095 cm^−1^ and ~636–613 cm, assigned to the O=S=O stretching and bending vibrations and S–O stretching vibration of the sulphonic acid (–SO_4_^2−^) groups of sulphate-based crystallites in accordance with the IR spectra of pure sulphate salts, being accompanied by a decrease in the –OH surface groups, confirming coverage of the CNF surfaces by them. It can also be observed that the presence of a small shoulder for the −OH groups (at ~3337 cm^−1^) in the case of samples incubated in MgSO_4_, MnSO_4_ and K_2_SO_4,_ were still present, indicating their availability, while it was not detected anymore for Na_2_SO_4_ due its full coverage followed by Na_2_CO_3_ and KOH, followed by the trends of the ions’ diffusion and swelling limitation of the CNF membranes in such electrolytes (Figure 3). The IR spectra for the representative CNF/MXene hybrid membranes, presented in Figure 5, followed the same trend, although they also showed significant differences in chemical structure between the upper and bottom sides after long-term incubation (i.e., several days of electrochemical performance under CV) in alkaline and monovalent electrolytes (KOH and Na_2_CO_3_). This might be due to even poorer/limited accessibility (rather than smaller penetration) of solvated ions to the anchoring sites on the CNF surface into the morphologically more closed structure on the bottom (freezing-exposed during preparation) side of the membranes prepared at a lower (kinetically faster) freezing temperature (−80 °C). The lowering of the intensities and shifting of the −OH related peaks (centred at ~3334 cm^−1^ and ~1030 cm^−1^) on the upper sides and peak intensification for both cations (K^+^ and Na^+^) and anions (CO_3_^2−^) for membranes in those electrolytes (Figure 5a,b), additionally confirming the involvement of these groups in the H-bonding with cations, covered the membrane surface fully by forming an uniform electroactive layer at the membrane–electrolyte interfaces after their further conjugation with anions, as already established by the results presented in Figure 2b and Figure S5. The −COOH groups present on the CNFs’ surfaces allow additional electrostatic interactions with the electrolyte cations (Figure 2). On the other hand, the membranes in the acidic medium did not show any particular chemical differences (Figure 5c,d), but became fully covered with the electrolyte layer on both membrane sides. The strongly intense peak at ~3200 cm^−1^ for the membrane tested in MgSO_4_ is due to the high hydroscopicity of Mg ions, forming different crystallites after anchoring with the SO_4_^2−^ anions.

Such results are also in line with the structural analysis supported with SEM-EDX (Figure 2), as well as chemical analysis performed with XRD and XPS (Figure 3), proving the structurally different integrity of electrolytes within the CNF matrix and their intercalations. As is evident from Figure 2, after the incubation of the pure CNF membrane sample in alkaline electrolyte solutions (1M KOH and 1M Na_2_CO_3_), the C content was reducing much more significantly than O, resulting in the reduction of the C/O ratio from ~1 to ~0.48 (KOH) and to ~0.28 (Na_2_CO_3_), while showing the presence of a similar quantity of cations (~38 wt% of K^+^, ~35 wt% of Na^+^), thus confirming the coverage of CNF with electrolytes formed into different crystallite structures. For the CNF-based membranes containing MXene, the content of both Ti and C was reduced significantly for the sample tested in KOH (from ~23 wt% to ~5 wt% for Ti, and from ~25 wt% to ~13 wt% for C) and slightly in Na_2_CO_3_ (to ~16 wt% and ~10 wt%, respectively), while F was not detected anymore and the content of O increased from ~29 wt% to ~46 wt% and 52 wt%, also confirming the structural changes of Ti_3_C_2_T_x_ MXene after incubation in such a highly alkaline medium. Indeed, different from the pure CNF membrane (Figure 2a), the CNF/MXene membrane (Figure 2b) had an almost completely covered surface with a salt-specific crystal structure (represented by XRD peaks of very high intensities, Figure 3a), formed on the interphase of both components at their anchoring sites. The broader peak between ~10 and 30° also indicates that the original crystal structure of cellulose was still retained. The XPS spectra (Figure 3b) reconfirmed the presence of C, O, Ti, and F atoms for the CNF/Ti_3_C_2_T_x_ membrane. The deconvoluted Ti 2p spectra for the same composite membrane exhibited a new peak of Ti-O at ~461.8 eV as compared to the Ti_3_C_2_T_x_ sample after mixing with CNF, interacting with the surface-decorated −OH and rare −COOH groups of the CNF [2], which, however, disappeared or shifted to ~460.5 eV after long-term incubation within KOH or Na_2_CO_3_, respectively. An intensely increased shoulder peak at ~463 eV in the Ti 2p spectra bound to TiO_2_ compounds also confirmed a kinetically faster formation of oxygenated species in KOH than Na_2_CO_3_. However, the broadened C-O and C=O related peak at ~287.2 eV in the C 1s spectra of the CNF/Ti_3_C_2_T_x_ membrane were reduced completely after long-term incubation in both alkaline electrolytes, and new peaks appeared at the binding energy value at ~292 eV and ~295 eV corresponding to the KOH, and at ~289 eV, which, with a peak at ~532 eV in the O 1s spectra, corresponded to the -CO_3_ surface decorated groups, thus confirming the formation of an electroactive layer on the surfaces. The H-bonding was again confirmed to improve the stability by enhancing interfacial adhesion between components of such hybrid membranes. From both the SEM and optical analysis, it is also evident that the black colour of MXene was lightening and the membranes yellowing, confirming distinctive changes of their physical and chemical characteristics, above all, the formation of TiO_2_ compounds.

### 2.4. Electrochemical Properties of Composite Membranes in Three-Electrode System

The ions’ diffusion and the capacitive behaviour of Ti_3_C_2_T_x_ MXene is influenced by the interlayer spacing (its expansion) and cation migration barrier, above all hydrophilic and negatively charged oxygen termination groups such as =O and −OH [43] on the MXene surface, which creates an electrostatic attraction with opposite charges in an acidic medium, and, thus, depending on the hydrodynamic size of the electrolyte cations, enables their adsorption and passage through the layers [28] to generate pseudocapacitance [49,50]. The high mass loading of active materials is thus crucial in achieving high capacitance (C) and energy density (E), as the energy density is evaluated at an applied voltage (V) by *E* = 1/2 CV^2^. However, besides the MXene surface chemistry (the presence of −F, −O, −OH) and stability after delamination, trace impurities (above all the partial removal of Al and presence of TiO_2_ and AlO_3_) in the nanofibrilated cellulose acting as a binder can thereby reduce the accessible area for ionic transport significantly, and, thus, the area next to their interactions, limiting the utilisation of the active sites of Ti_3_C_2_T_x_ MXene and reducing its electrochemical properties [5]. The size of the hydrated cations and their interaction with dissociated functional groups on CNFs that have to match the electroactive site (Ti_3_C_2_T_x_ MXene) in such a hybrid electrode material are thus also crucial, resulting in limited capacity values. The larger ions that cannot penetrate can, therefore, only contribute to the cyclic stability of the intercalation layers by forming EDL capacitance by electrochemical adsorption [48].

Since the CNF/MXene membrane’s physico-chemical properties (swelling/shrinkage) and stability are highly dependent on the environmental pH (Figure 4b), electrochemical measurements of the CNF/MXene membranes prepared with 50 wt% Ti_3_C_2_T_x_ MXene at both freeze-casting temperatures (−50 °C and −80 °C) acting as a cathode in a three-electrode setup were investigated using pH and ions-type different aqueous electrolyte solutions. Membranes prepared with higher (90 wt%) MXene content gave surprisingly comparable capacitance values when tested in the generally used H_2_SO_4_ and KOH (Figure S6) but demonstrated poorer mechanical stability (Figure S1). Therefore, they were not selected for further testing. First, cyclic voltammetry (CV) experiments were conducted for different electrolytes to determine their voltage window (vs. Ag/AgCl) and to understand the capacitive behaviours (gravimetrical, areal and volumetric) of CNF/MXene-based electrodes at different scan rates and time intervals. The CV curves (presented in Figures S7 and S8) showed very similar profiles (redox peaks) by increasing the scan rates, suggesting that the electrodes have a relatively fast ionic response. One broad peak at the cathode and one broad at the anode were observed for all pH slightly acidic electrolytes, which corresponded to the insertion and extraction reactions of specific cations (H^+^, K^+^, Na^+^, Mg^2+^) during cycling (charging and discharging). The charge and discharge curves had no visible plateau, indicating that the capacitance originates mainly from the pseudocapacitance, i.e., reversible intercalation/deintercalation of protons due to the change of the Ti oxidation state, which, as expected, was most exposed for the hydrodynamically smallest and fastest H^+^ cations using H_2_SO_4_ [49,50]. According to the surface area of CV curves, the pseudocapacitance generated by other electrolyte (larger) cations in a such a slightly acidic sulphated media was much smaller due both their hydrodynamically limited mobility to and through the MXene layers, as well as interactions with the CNF surfaces additionally stabilized by sulphate anions. On the other hand, the weak cathodic peak, ranging from 0.5 to 0.6 V and from 0.9 to 1.1 V for basic electrolytes (KOH and Na_2_CO_3_, respectively), can be attributed to the formation of an electroactive layer on the interface (as confirmed by the other studies and observed well from the SEM images presented in Figure 2), which was increasing for the subsequent cathodic scans, due to the intercalation of K^+^ and Na^+^ ions to/into the Ti_3_C_2_T_x_ that formed Ti_3_C_2_T_x_K_x_/Ti_3_C_2_T_x_Na_x_. In the anodic scans, the broader peak between 0/0.2–0.4 V and 0.2–0.7 V was assigned to the extraction of cations from the Ti_3_C_2_T_x_ MXene, being wider and larger for the membranes prepared at −80 °C.

**Figure 6:**
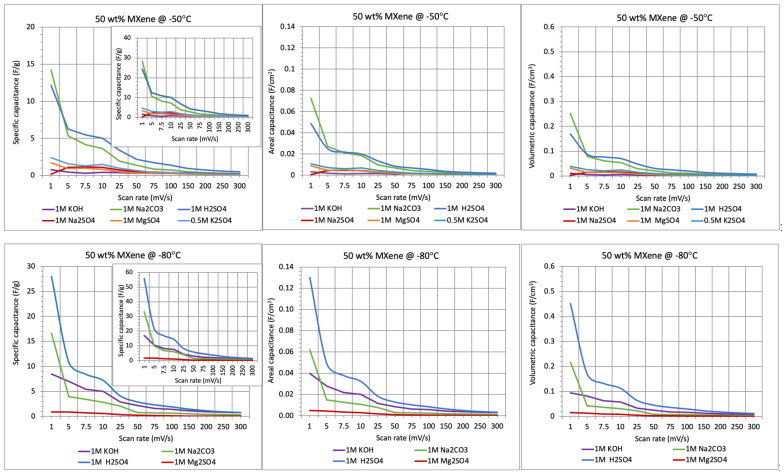
Specific (F/g), areal (F/cm^2^) and volumetric (F/cm^3^) capacitances for CNF-based membranes containing 50 wt% of Ti_3_C_2_T_x_ MXene (HT-etched), prepared at −50 °C vs. −80 °C, depending on the electrolyte type and scan rates. The inserts show the gravimetric capacitance of the electrodes based on the Ti_3_C_2_T_x_ weight.

However, the mass-specific (gravimetric) capacitance of the CNF/MXene membranes was generally very low, and it decreased additionally with the increasing scan rate, as illustrated in Figure 6. As already established in other studies [5] and reconfirmed in this one, as discussed above, such low capacitance is due to the presence of traces of Al and formed oxidised species (TiO_2_ and AlO_3_) on the surface of the Ti_3_C_2_T_x_ MXene layers, which affects the inhomogeneous and less open interlayer structures, and thereby reduces the interactions of the electrolyte cations with the terminal groups on the MXene (=O, –OH, and –F), limiting their penetration to transport through the MXene nanosheets and thus its electrochemical ability. The accessibility of ionic transport is additionally reduced due to interactions of cations with the negatively charged functional groups (–OH, and –COOH) on the CNF’s outer surface, highly depending on the electrolyte pH, size and valence of hydrated cations, as discussed in previous section. Relatively low measured specific capacitance values were thus obtained for the highly alkaline KOH electrolyte, and even lower for all the sulphate electrolyte solutions, except in highly acidic H_2_SO_4_ as well as medium alkaline Na_2_CO_3_ (Figure S7). At higher current rates, the capacitances additionally decreased significantly, showing still higher behaviour in H_2_SO_4_ and Na_2_CO_3_ electrolytes, and using a denser membrane prepared at −80 °C. At a low scan rate (~5 mV/s), the membrane capacitance for the H_2_SO_4_ electrolyte solution thus showed more than ~66% higher values (~10 F/g) for the membrane prepared at −80 °C as compared to that at −50 °C (~6 F/g), or using Na_2_CO_3_ as the electrolyte (6–4 F/g), due to both the higher (not interacting with CNFs) ionic mass transfer ability of the hydrodynamically smaller (H^+^) cations to the active sites on the Ti_3_C_2_T_x_ interfaces and their reduced diffusion path in the case of the denser membrane, which increased both the kinetics and capacitance. This advantage cannot be utilised at higher scan rates due to still-limited access to the interlayer spacing in MXene for larger ion-accessible surface areas, favouring faster ion transport. The excessive amounts of electrolyte in the electrode pores which blocks them (in the case of acidic sulphate electrolytes) or layered structure formed on the membrane interface (in the case of basic electrolytes) impedes the ions’ mobility additionally, and, thus, accessibility to active sites on the Ti_3_C_2_T_x_. The normalised gravimetric capacitances (presented by the inserted graphs), calculated solely based on the weight of Ti_3_C_2_T_x_ using the composition ratios of the initial hybrid dispersions, increased almost once (~100%, to ~21 F/g and ~12 F/g, respectively) for these two electrolytes (including KOH for the membrane prepared at −50 °C) at all scan rates, additionally confirming the effect of microstructural composition of the CNF/Ti_3_C_2_T_x_ MXene membranes and their interactions with electrolytes, that limits mass transport. It can also be concluded that, compared to highly acidic H_2_SO_4_, the selected slightly acidic electrolytes with monovalent cations (e.g., K_2_SO_4_ and Na_2_SO_4_) display much lower capacitance (~25–17% at 5 mV/s), but still higher than that with divalent Mg^+^ (MgSO_4_, ~7%), while exhibiting a similarly high current response in a less alkaline electrolyte with monovalent Na^+^ (Na_2_CO_3_, ~85/50%) for the composite membrane prepared with 50 wt% of MXene at −50/80 °C. Although the gravimetric capacitance was not comparable to some of the currently reported NC/MXene-based SCs, summarized in Table S1, the areal and volumetric performance values were quite similar, which can be due to the shrinkage of membranes across their surface area and/or thickness (Figure 4) in all electrolytes, which causes stacking of diffused ions and limits mass transfer.

**Figure 7:**
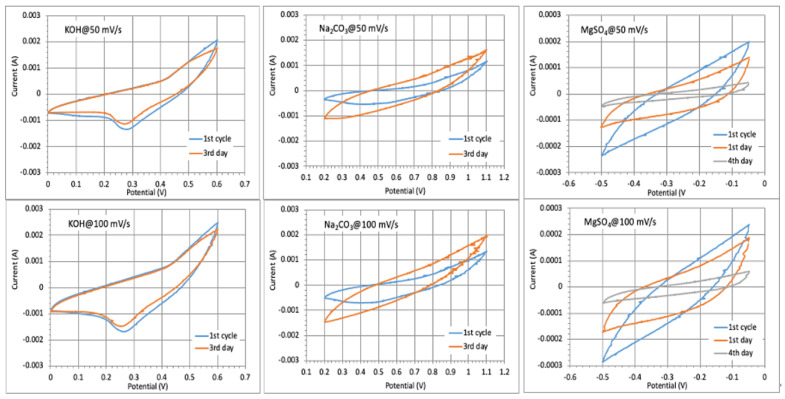
Cyclic voltammogram (CV) curves at scan rates of 50 and 100 mV/s for selected electrolytes using a CNF-based electrode (1 cm^2^) prepared with 50 wt% Ti_3_C_2_T_x_ MXene (HT-etched) at −80 °C, after up to several days of cycling.

**Figure 8:**
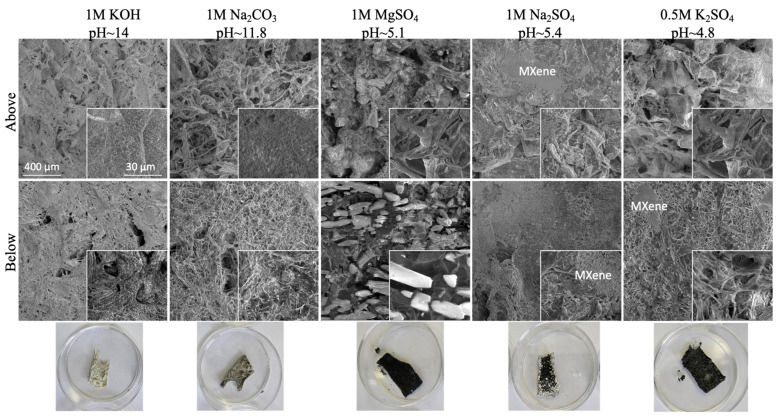
SEM images and photographs of selected CNF-based electrode surfaces prepared with 50 wt% Ti_3_C_2_T_x_ MXene (HT-etched) at −80 °C after a few days of cyclic voltammetry (CV) testing at scan rates of 50 mV/s in different electrolytes.

The repeated cyclic performance of the electrode prepared at −80 °C in different electrolyte solutions (Figure 7) showed almost a 100% CV curve profile after a few days cycling in KOH (comparatively lower electrolyte uptake with intensive membrane shrinkage, Figure 4b), a slight enlarging of voltammograms in Na_2_CO_3_ (increased adsorption ability of the monovalent cations with slight membrane swelling), and reduction in MgSO_4_ (the lowest diffusion ability of larger Mg^2+^), while the long-time testing could not be performed in H_2_SO_4_ due to membrane instability (CNF degradation) in such a highly acidic medium, as already discussed above (Figure S3). However, the capacitance values were reduced to ~75% after 1000 numbers of cycling in KOH due to the slow oxidation of MXene to TiO_2_ in such a highly basic electrolyte solution (pH 14), as was also evident from the sample whitening (the photograph presented in Figure 8), while the sample remained quite stable in Na_2_CO_3_ and retained 90% capacitance. During the first few cycles, the electrolyte reached the active sites easily, and the capacity increased accordingly. Considering that CNF has no electrochemical capability, the changing of voltammogram areas after charging–discharging is thus related to the accessibility or limitation of active sites at the Ti_3_C_2_T_x_ for transport of differently large ions due to the interaction with CNFs, their swelling/shrinkage ability in an electrolyte solution and pH-dependent electrostatic attraction interactions with the cations [37]. These results further show that the type of CNFs and their interactions with electrolyte ions thus have a significant impact on the rate and capacitance of CNF/Ti_3_C_2_T_x_ electrodes, i.e., the accessibility to surface active sites on Ti_3_C_2_T_x_ to store the ions after redox reactions, exhibiting a pseudocapacitive property, which, however, is also significantly dependent on the type and size of the cations.

## 3. Conclusions

Flexible free-standing membranes were prepared from different weight percentage ratios of cellulose nanofibrils (CNFs) and 2D Ti_3_C_2_T_x_ MXene (10:90, 50:50, 90:10) as electroactive materials through the deep freeze-casting method (at −50 °C and −80 °C), to evaluate their physico-chemical properties and long-term stability in different (strong/slightly acidic and basic) electrolyte solutions, and thereby examine their impact on the process of charging and discharging and overall pseudocapacitance when used as electrodes in a three-electrode setup configuration. The problem of using highly acidic electrolytes (such as H_2_SO_4_, pH~0.35) is due to the CNFs’ degradation, as well as highly basic electrolytes (as KOH, pH~14), due to fast Ti_3_C_2_T_x_ MXene oxidation being confirmed to redox inactive TiO_2_. The pH-dependent charge and swelling of such electrodes and available surface termination groups on both CNF and MXene, next to an inhomogeneous and unstable interlayer MXene structure due to the presence of traces of aluminium and formed oxides on its surface, was shown to affect its capacitance behaviour. It was demonstrated that the kinetics and capacitance of membranes in basic electrolytes were predominantly surface-controlled, due to the CNFs’ surface interaction with electrolyte cations via ionised negative surface functional groups and membrane shrinkage, leading to the formation of an electrolyte layer, which increases their cyclic stability, but limits the cations’ diffusion to the MXene interface and their irreversible capacity; this effect was still less pronounced for a thinner and more dense membrane prepared at −80 °C and 50 wt% of MXene content. The charge and discharge curves in the cycling voltammograms also demonstrated that the capacitance comes mainly from the pseudocapacitance (electrochemical redox reactions), being associated with the reversible intercalation/deintercalation ability of cations to the active sites into multi-layered Ti_3_C_2_T_x_ MXene, and more exposed in slightly acidic electrolytes (Na_2_SO_4_, MgSO_4_, MnSO_4_, K_2_SO_4_), while, however, still being limited by the ions’ hydrodynamic size (K^+^, SO_4_^2−^ > Na^+^ > Mn^2+^ > Mg^2+^), their interacting and stacking during diffusion and membrane swelling; divalent cations were shown to be more favourable for the adsorption on the CNF than monovalent cations, among which the smaller and highly conductive K^+^ ions diffuse and interact faster than Na^+^, and both much faster/easier than Mn^2+^ and Mg^2+^. Although the obtained capacitances in the most suitable (still less aggressive) electrolyte solution (Na_2_CO_3_) were typically too low (8 F/g, 0.04 F/cm^2^, and 0.03 F/cm^3^ at 2 mV/s) to be of practical importance, such a low-cost membrane shows rechargeability and excellent cycling stability (90% retention after three days of cycling). To increase the electrochemical performance, the membrane’s structure should be optimised further by constructing a more homogeneous and preferably nanoporous architecture, potentially using thinner and more size-homogeneous CNFs, surface functionalised or modified in a way that would, with appropriate crosslinking and weight ratio, mitigate the ion-blocking effect and shorten the cations’ diffusion paths to the MXene interfaces, and, thus, increase the kinetics and capacitance, while maintaining mechanical flexibility.

## 4. Materials and Methods

### 4.1. Materials

Cellulose nanofibrils (CNFs) with diameters of 10–70 nm and lengths of a few (1–3) μm, were prepared from bleached softwood pulp at the University of Maine (USA), The Process Development Center. The surface charge of around −32.6 mV was determined by Dynamic Light Scattering analysis using a Zetasizer (Nano ZS ZEN360, Malvern Instruments Ltd., Malvern, UK), being related to the presence of rare (~0.12 mmol/g) carboxylic (–COOH) groups, as evaluated by potentiometric titration (Mettler Toledo T-70 equipped with a combined glass electrode Mettler TDG 117, Metter Toledo d.o.o., Dobrunje, Slovenia), respectively. The titanium aluminium carbide (Ti_3_AlC_2_) powder (MAX phase), of 10–30 μm in a diameter, was purchased from Nanochemazone (Alberta, Canada). All the chemicals and solutions were purchased from Sigma-Aldrich GmbH, Darmstadt, Germany, and used as received.

### 4.2. Preparation of Ti_3_C_2_T_x_ (MXene) from Ti_3_AlC_2_ (MAX Phase)

Two different etching routes were used to produce Ti_3_C_2_T_x_ [51,52,53]. For the HF route, 0.5 g of Ti_3_AlC_2_ powder was added gradually in a course of five minutes to 10 mL of 30% hydrofluoric acid (HF) while stirring with magnetic bar at 20 rpm and continued with treatment for 24 h. For the LiF-HCl route, 0.5 g of Ti_3_AlC_2_ powder was dispersed in 10 mL of 9M hydrochloric acid (HCl) and 12M lithium fluoride (LiF) by magnetic stirring at 20 rpm and continued to treat for 24 h. Both processes were performed at room temperature. Thereafter, the samples were washed several times (~40) by adding 50 mL of deionised water in a graduated conical tube, and centrifugation (10 min per cycle at 3500 rpm using a Rotina 380R centrifuge; Hettich, Kirchlengern, Germany) until the supernatant pH value reached ~6. The Ti_3_C_2_T_x_ sediments were rewashed with 1000 mL of deionised water via vacuum-assisted filtration, using a polyvinyl difluoride (PVDF) filter membrane with a 0.22 μm pore size (Durapore, Merk Millipore, Massachusetts, USA) before collection of the remaining materials. The prepared samples were further intercalated/delaminated by magnetic stirring at 20 rpm for 24 h in dimethylsulphoxide (DMSO) (99.9%), followed by 1 h sonication at 20% amplitude using a Vibra Cell VCX 750 sonicator (Sonics, Newtown, CT, USA). The obtained MXene powders were dried in a vacuum at 100 °C.

### 4.3. Fabrication of Composite Membranes

A total of 15 mL of 0.69 wt% of water-suspended CNF was prepared. At the same time, various quantities (0.01, 0.1035 and 0.9315 g) of Ti_3_C_2_T_x_ MXene were dispersed homogeneously in 15 mL milliQ water by ultrasonic processor Vibra Cell VCX 750 (Sonics, USA) for 1 h at 20% amplitude. Finally, each of the Ti_3_C_2_T_x_ dispersions was mixed with the CNF suspension and stirred again for 1 h to obtain homogeneous CNF/Ti_3_C_2_T_x_ dispersions with a final 10 wt%, 50 wt% and 90 wt% of Ti_3_C_2_T_x_. The as prepared CNF/Ti_3_C_2_T_x_ dispersions were transferred to polystyrene Petri dishes (Greiner Bio-one, Kremsmünster, Austria) of 90 mm in diameter, and placed on a temperature-controlled (by the software program Supercool^®^) Cu-plate of a self-constructed cryo-unit, with temperatures set to −50 °C and −80 °C, respectively, for about 30 min, to allow for the cryogelation process. The prepared membranes were lyophilised for 48 h at a temperature of about −50 °C and pressure of 0.5 mmHg, using Mini Lyotrap Freeze Dryers provided by LTE Scientific (Oldham, UK), to sublime all the ice crystals of the water being generated during the freezing process. The pristine CNF membranes without Ti_3_C_2_T_x_ MXene addition were prepared in the same way.

### 4.4. Characterisation

Dimensional changes and their uptake have been studied in different electrolyte solutions. Due to different saturation in the solubility of electrolytes at room temperature, 1M concentrated solutions were used, except for K_2_SO_4,_ where 0.5 M was taken. The dimensional changes (in surface area/SA (Equation (1)) and thickness/T (Equation (2))) of the membranes were analysed by soaking them for 24 h or 7 days in different electrolyte solutions at room temperature. For this purpose, their dimensions (*SA*, cm^2^) and thicknesses (*T*, cm) were measured before (*SA_dry_* and *T_dry_*) and after (*SA_wet_* and *T_wet_*) immersion. In addition, the electrolyte solutions’ uptake (*W*, mg, Equation (3)), wet areal (ρ_A_, g/cm^2^), and volumetric (ρ_V_, g/cm^3^) densities of the samples were provided, and the porosity (*ɛ*) calculated by Equation (4).

Surface area/*SA* change = (*SA_wet_* − *SA_dry_*)/*SA_dry_* × 100 (%) (1)

Thickness/*T* change = (*T_wet_* − *T_dry_*)/*T_dry_* × 100 (%) (2)

Electrolyte solution uptake (weight, *W*) = (*W_wet_* − *W_dry_*)/*W_dry_* × 100 (%) (3)

Porosity/*ɛ* = 1 − density × 100 (%) (4)

The presented results are mean values with Standard Deviations of at least two independent measurements.

SEM imaging was performed, to evaluate the samples’ morphology and elemental composition by using a low-vacuum Scanning Electron Microscope, FEI Quanta 200 3D (Thermo Fisher Scientific Inc., Waltham, MA, USA), equipped with INCA 350 Energy Dispersive X-ray spectrometer (EDX; Oxford Instruments Ltd., Abingdon, UK).

Different spectroscopic methods were used to evaluate the transition of the MAX phase to the MXene, as well as to characterise the chemical interactions between the membrane components and the electrolyte ions on the surface and in the bulk phase, and to evaluate their crystallinity.

The Fourier Transform Infrared (FTIR) spectra were recorded by using a Spectrum One spectrometer (Perkin-Elmer Inc., Shelton, CT, USA). The transmission measurements were carried out in the range of 400–4000 cm^−1^ with 16 scans and a resolution of 4 cm^−1^. The Spectrum 5.0.2 software program (version 10.6.1) was applied for the data analysis. Few measurements were made per film.

The X-ray Photoelectron Spectroscopy (XPS) measurements were performed with a Supra plus device (Kratos Analytical Ltd., Manchester, UK), equipped with an Al Kα excitation source. The charge neutraliser was on during the spectra acquisition. The binding energy scale was corrected using the C–C/C–H peak at 284.8 eV in the C 1s spectra. The samples were attached to the sample holder using carbon tape. The measurements were carried out at a 90° take-off angle, at a pass energy of 20 eV and a step of 0.1 eV. The analysis area was 400 μm. The spectra were measured and processed using the ESCApe 1.5 software (Kratos Analytical Ltd., Manchester, UK).

The X-ray Diffraction (XRD) patterns of the MAX phase, MXene and composite membranes, were obtained with a 2D phaser X-ray diffractometer (Bruker AXS GmbH, Karlsruhe, Germany) using Cu Kα radiation (λ = 0.15184 nm) at the operating voltage and current of 30 kV and 10 mA, respectively, at room temperature (23 ± 1 °C) within a 2θ value ranging from 5 to 80° and an increment of 0.03°. A silicon sample holder with a zero background was utilised in all the tests.

Thermogravimetric analysis (TGA) was performed in addition, to evaluate Al removal from the MAX phase using a Differential Scanning Calorimeter (DSC1, Mettler Toledo GmbH, Greifensee, Switzerland), under a nitrogen atmosphere in a temperature range from 25 to 600 °C and a heating rate of 10 °C/min using a 50 mL/min flow rate.

### 4.5. Membranes’ Electrochemical Measurements

The Cyclic Voltammetry (CV) measurements were carried out in a three-electrode configuration, by using the PGSTAT101 Autolab controlled by the Autolab Nova Software, version 2.1.6. The CV experiments were carried out in a 90 mL glass titration vessel filled with 50 mL of the selected electrolyte solution, and with a 1 cm^2^ area of membrane sample acting as a working electrode exposed to the electrolyte solution. A platinum ring was used as a counter, and Ag/AgCl electrodes as the reference electrodes (both provided from Metrohm, Primalab d.o.o. Slovenia). The measurements were carried out at room temperature. The electrochemical capacitance was evaluated by the following Equations:

(5) $$\mathrm{Specific}\;\mathrm{capacitance}\;(F/g):\;C=\frac{1}{m∆Vk}\int_{v-}^{v+}I\;dV$$

(6) $$\mathrm{Areal}\;\mathrm{capacitance}\;(F/{\mathrm{cm}}^{2}):\;C=\frac{1}{a∆Vk}\int_{v-}^{v+}I\;dV$$

(7) $$\mathrm{Volumetric}\;\mathrm{capacitance}\;(F/{\mathrm{cm}}^{3}):\;C=\frac{1}{u∆Vk}\int_{v-}^{v+}I\;dV$$

where *m*, *a* and *u* represent the mass (g), the surface area (cm^2^) and the volume (cm^3^) of the electrode material (CNF/MXene membrane sample), respectively, ∆*V* is the applied potential difference, and *k* is the scan rate (mV/s).

## Figures and Tables

**Table 1:** The surface and bulk/volumetric densities and total porosity, obtained by gravimetric analysis, for freeze-dried CNF-based membranes prepared with and without the addition of MXene (HF-etched) at different freezing temperatures.

Sample Preparation	MXene Addition (wt%)	MXene Loading (mg/cm^2^)	Areal Density, *ρ_A_* (mg/cm^2^)	Bulk/Volumetric Density, *ρ_V_* (mg/cm^3^)	Porosity, *ɛ* (%)
−50 °C	0	0	5.4 ± 0.05	1.8 ± 0.02	99.81
	10	0.25 ± 0.01	15.9 ± 0.8	5.3 ± 0.1	99.47
	50	1.9 ± 0.3	21.2 ± 1.2	6.5 ± 0.3	99.35
	90	15 ± 1.0	82.9 ± 2.5	14.1 ± 0.9	98.59
−80 °C	0	0	6.7 ± 0.08	2.5 ± 0.05	99.64
	10	0.28 ± 0.02	17.6 ± 0.9	7.1 ± 0.1	99.29
	50	2.5 ± 0.5	22.2 ± 1.1	7.7 ± 0.1	99.23
	90	16 ± 1.2	82.4 ± 2.9	19.1 ± 1.2	98.09

## Data Availability

The original contributions presented in this study are included in the article/Supplementary Material. Further inquiries can be directed to the corresponding author.

## Supplementary Materials

The following supporting information can be downloaded at: https://www.mdpi.com/article/10.3390/gels11040265/s1, Table S1: Comparison of the electrochemical capacitance properties of different NC/Ti_3_C_2_T_x_ MXene-based composite (film/aerogel/cryogel) electrodes performed by CV. References [2,19,37,38,39,40,41,45] are cited in the Supplementary Materials. Figure S1: The FTIR spectra of the membranes prepared with 50 wt% Ti_3_C_2_T_x_ MXene at different freezing temperatures (−50 °C and −80 °C) and surface side, compared to solvent-casted films and dried CNFs. Figure S2: Photos of CNF-based membranes prepared without and with 10/50/90 wt% Ti_3_C_2_T_x_ MXene at −80 °C after 24 h and 7 days of immersion in different electrolyte solutions. Figure S3: Photo and SEM images for membranes prepared with and without 50 wt% Ti_3_C_2_T_x_ MXene at −80 °C, after 7 days immersion in H_2_SO_4_ (pH~0.35). Figure S4: Characterisation of CNF/Ti_3_C_2_T_x_ MXene (HF-etched) membranes prepared at different freeze-casting temperatures (−50 °C vs. −80 °C), after 24 h of immersion in different 1/0.5M electrolyte solutions: (a) The uptake of electrolyte solutions, (b) The swelling/shrinkage percentage based on surface area (SA, cm^2^) and thickness (T, cm) change, (c) The wet volumetric density (ρ_V_, g/cm^3^) and total porosity (ɛ) change, (d) The reduction of the solutions’ pH after 7 days of membrane incubation. Figure S5: Characterisation of pure CNF-based membranes (without MXene), prepared at −50 °C after 24 h of immersion in different electrolyte solutions: (a) The uptake, the swelling/shrinkage percentage based on surface area and thickness, and pH percentage reduction, (b) The wet volumetric density and total porosity change. (c) The FTIR spectra after 7 days of incubation in electrolytes, compared to the electrolyte powders. Figure S6: Specific, areal and volumetric capacitances at various scan rates for 1M H_2_SO_4_ and 1M KOH electrolytes, using membranes prepared with 10 wt%, 50 wt% and 90 wt% Ti_3_C_2_T_x_ MXene at −80 °C. Cyclic voltammogram (CV) curves at scan rates of 50 mV/s (Figure S7) and for various scan rates (Figure S8) for different electrolytes using membranes prepared with 50 wt% Ti_3_C_2_T_x_ MXene at different freeze-casting temperatures (−50 °C vs. −80 °C).
