# Supplementary material for: Electrochemical Capacitance of CNF–Ti3C2Tx MXene-Based Composite Cryogels in Different Electrolyte Solutions for an Eco-Friendly Supercapacitor
Source: Gels. 2025 Apr 3;11(4):265. doi: 10.3390/gels11040265 (PMC12026625; doi:10.3390/gels11040265)
Supplement: Supplementary file 1 [file gels-11-00265-s001.zip › gels-3510459-supplementary.pdf]

gels

MDPI

Article

Electrochemical Capacitance of CNF–$Ti_3C_2T_x$ MXene-Based Composite Cryogels in Different Electrolyte Solutions for an Eco-Friendly Supercapacitor

Vanja Kokol *, Subramanian Lakshmanan and Vera Vivo

Academic Editors: Hyun-Joong Chung and Xiaopeng Peng

Received: 18 February 2025
Revised: 28 March 2025
Accepted: 31 March 2025
Published: date

Citation: Kokol, V.; Lakshmanan, S.; Vivod, V. Electrochemical Capacitance of CNF—$Ti_3C_2T_x$ MXene Based Composite Cryogels in Different Electrolyte Solutions for an Eco-Friendly Supercapacitor. *Gels* **2025**, *11*, x. https://doi.org/10.3390/xxxxx

Copyright: © 2025 by the authors. Submitted for possible open access publication under the terms and conditions of the Creative Commons Attribution (CC BY) license (https://creativecommons.org/licenses/by/4.0/).

Table S1. Comparison of the electrochemical capacitance properties of different NC/$Ti_3C_2T_x$ MXene-based composite (film/aerogel/cryogel) electrodes performed by CV. Legend: vacuum-filtered (VF) film, vacuum-dried (VD), freeze-dried (FD) aerogel, ethylenediamine (EDA), bacterial cellulose (BC), cellulose nanofibrils (CNF), microfibrillated cellulose (MFC), reduced graphene oxide (rGO), porous carbon (PC), liquid metal (LM); *Capacitance capabilities normalized to the integrated MXene weight.

Sample (weight ratio)	Prepa-ration method	Capacitance at specific conditions	SC retained (%) after No. of cycles	Electrolyte used	Ref
$Ti_3C_2T_x$:BC (1.5:1)	VF	111.5 F/g, 0.112 F/cm^2 at 5 mV/s	100% 5000	PVA/H_2SO_4 gel	[45]
$Ti_3C_2T_x$:BC (5:1) (MXene: ~5 mg/cm^2)	FD	*416 F/g, 2.084 F/cm^2 at 20 mV/s	96.5 % 10000	3M H_2SO_4	[19]
$Ti_3C_2T_x$:carboxy-methyl-ated-CNF (4:1)	VF	298 F/g, 0.025 F/cm^2 at 2 mV/s	100% 10000	3M H_2SO_4	[37]
$Ti_3C_2T_x$+rGO:CNF (1:1)	FD	280 mAh/g at 100 mA/g	84.8 % 1000	1M $NaClO_4$	[40]
$Ti_3C_2T_x$:PC:CNF (1:1:1)	VF	94 F/g, 0.143 F/cm^2 at 0.1 mA/cm^2	n.d.	6M KOH	[2]
$Ti_3C_2T_x$:MFC (1:4.5)	Surface coating	451 F/g at 1A/g	n.d.	1M H_2SO_4	[39]
$Ti_3C_2T_x$:sulphated-CNF (not given)	VD FD	191 F/g 220 F/g at 1 A/g	100% 5000	1M H_2SO_4	[38]
$Ti_3C_2T_x$:LM:CNF (4:3:1)	VF	0.87 F/cm^2 at 5 mA/cm^2	96.9 % 2000	PVA/H_2SO_4 gel	[41]
$Ti_3C_2T_x$:CNF (1:1) (MXene: ~2.5 mg/cm^2)	FD (-80°C)	*50 F/g, 0.1 F/cm^2, 0.35 F/cm^3 at 2 mV/s	85.5 % 1000	1M H_2SO_4 CNF degrada-tion	This study
		*15 F/g, 0.04 F/cm^2, 0.17 F/cm^3 at 2 mV/s	90% 1000	1M Na_2CO_3	
		*8 F/g, 0.03 F/cm^2 0.08 F/cm^3 at 2 mV/s	75.5 % 1000	1M KOH MXene oxida-tion	

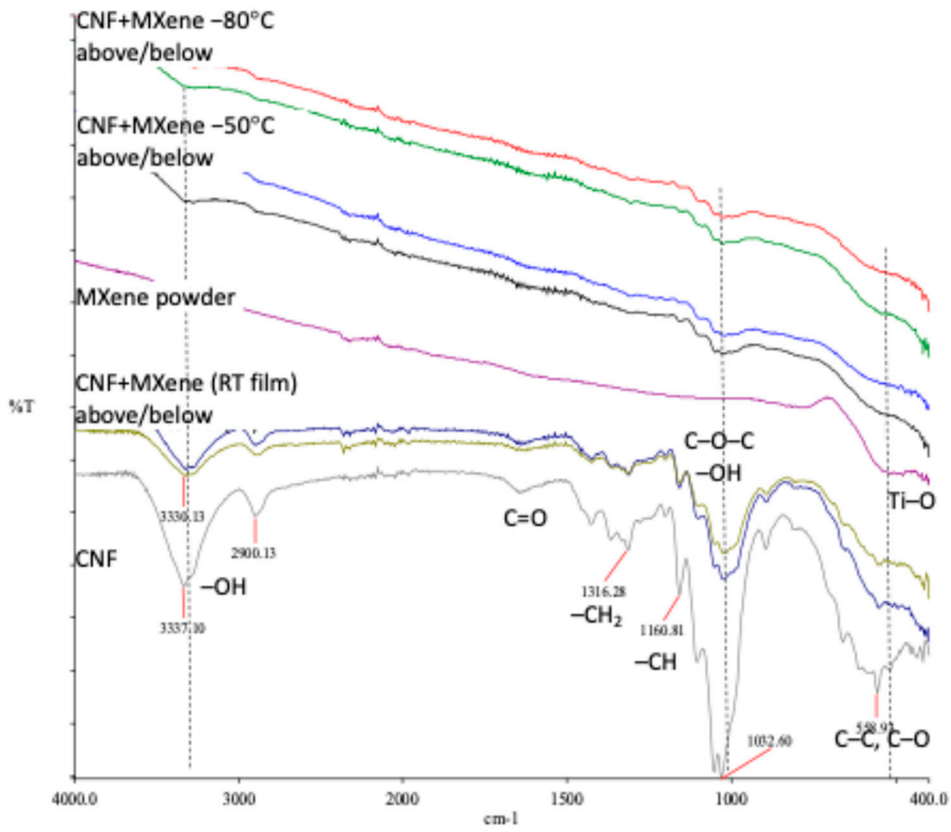

Figure S1. The FTIR spectra of the CNF-based membranes prepared with 50 wt% $Ti_3C_2T_x$ MXene (HT-etched) at different freezing temperatures (−50°C and −80°C) and surface side, compared to solvent-casted films and dried CNFs.

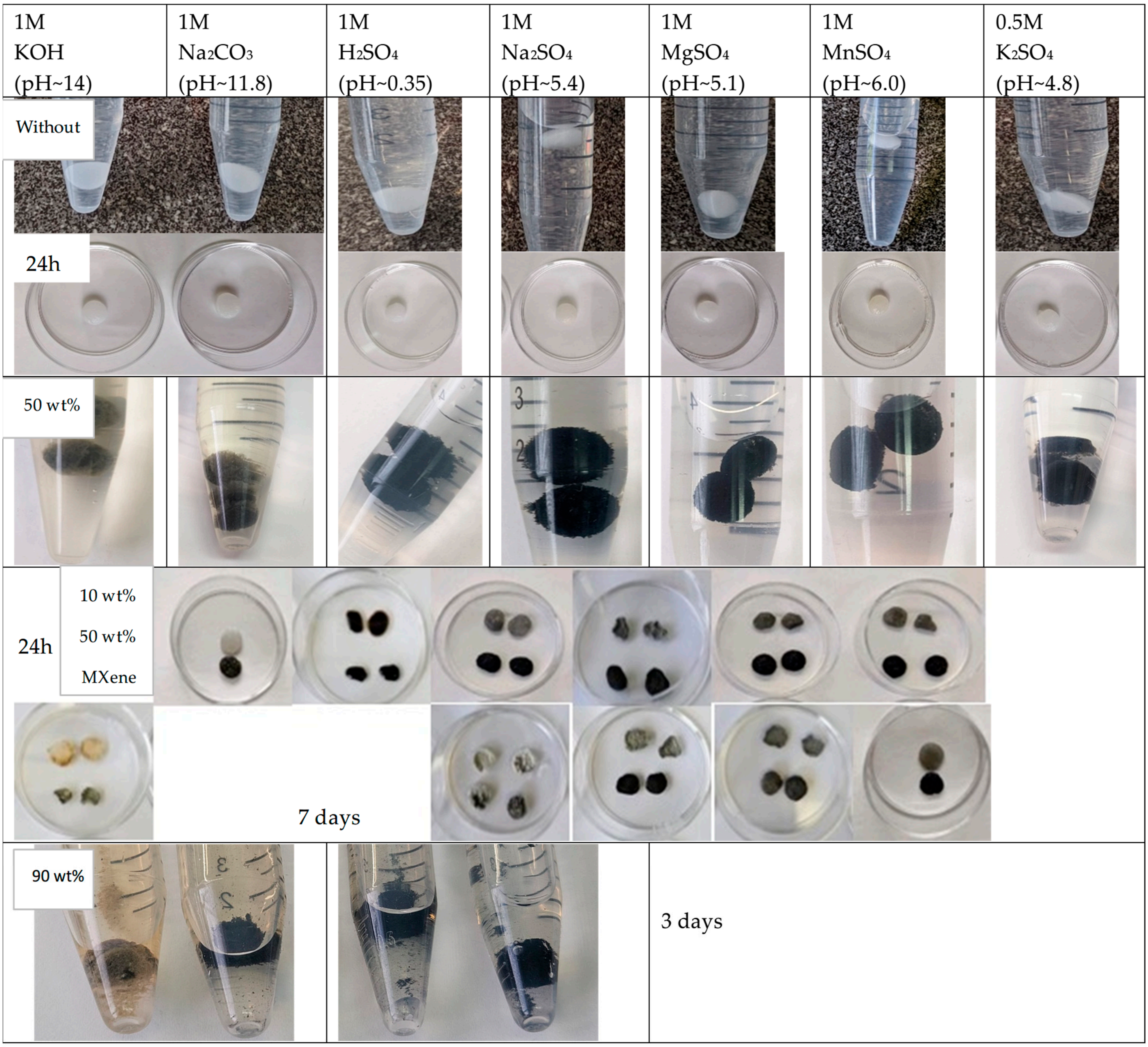

Figure S2. Photos of CNF-based membranes prepared with and without 10/50/90 wt% $Ti_3C_2T_x$ MXene (HT-etched) at -80°C after 24 h and 7 or 3 days of immersion in different electrolyte solutions.

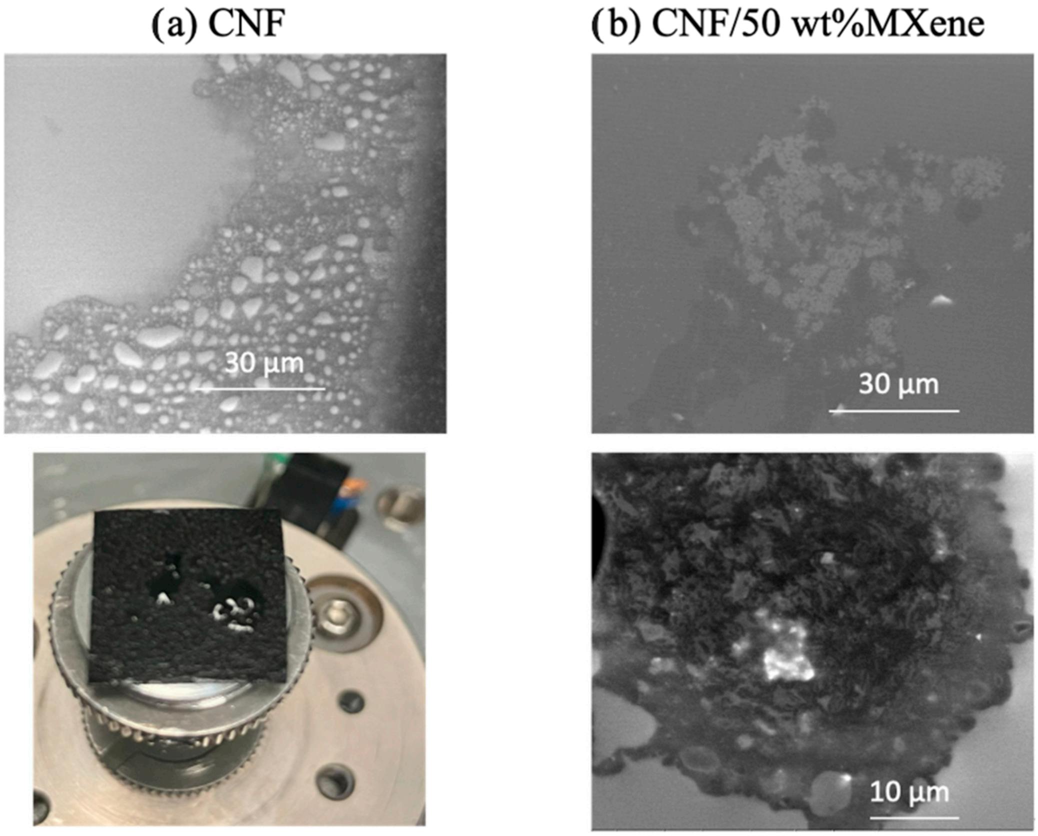

Figure S3. Photo and SEM images for CNF-based membranes prepared (**a**) without and (**b**) with 50 wt% $Ti_3C_2T_x$ MXene (HT-etched) at −80°C, after 7 days immersion in H_2SO_4 (pH~0.35) electrolyte solutions.

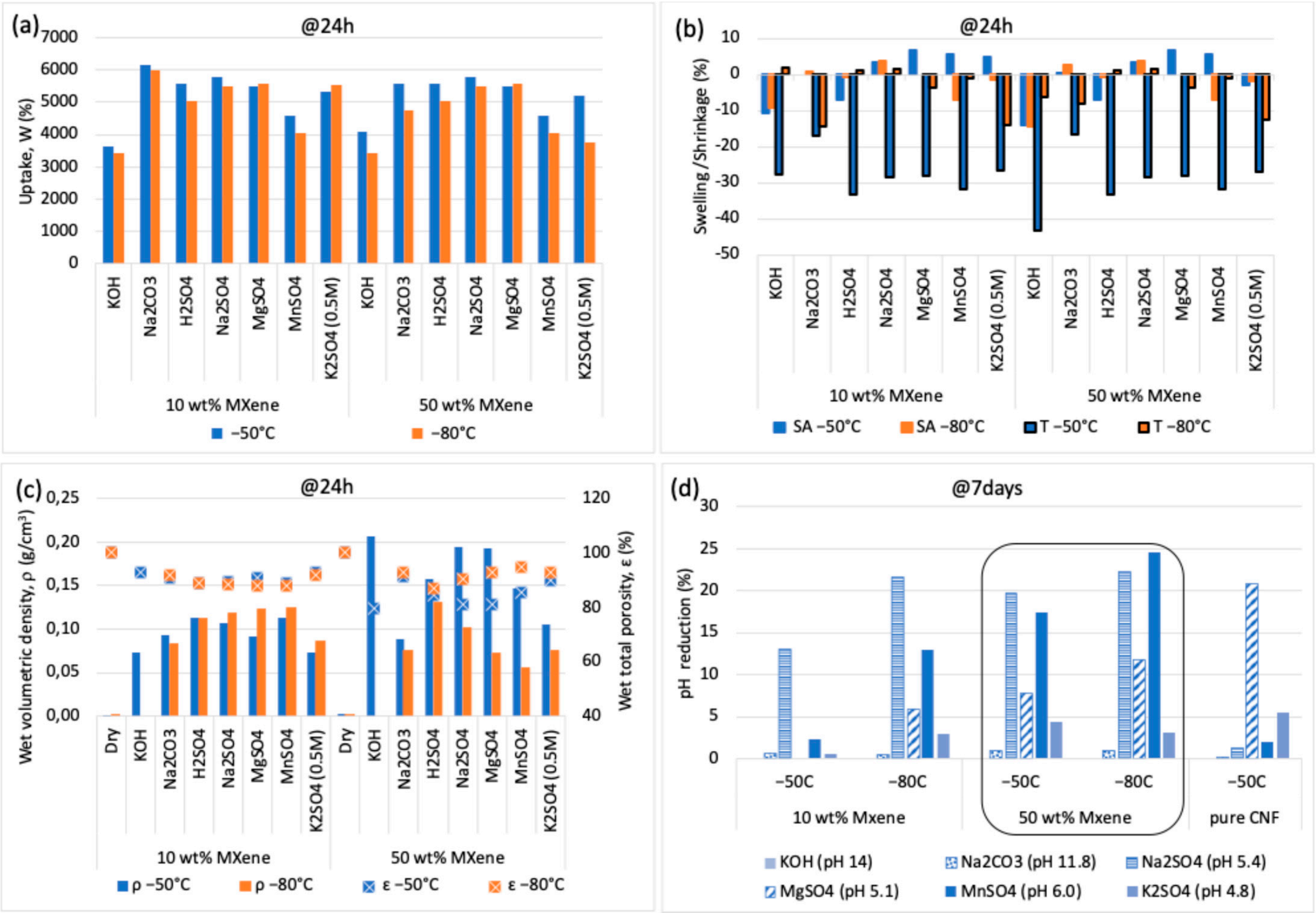

Figure S4. Characterisation of CNF/$Ti_3C_2T_x$ MXene (HF-etched) membranes prepared at different freeze-casting temperatures (−50°C vs. −80°C), after 24 h of immersion in different 1/0.5M electrolyte solutions: (**a**) The uptake of electrolyte solutions, (**b**) The swelling/shrinkage percentage based on surface area (SA, cm²) and thickness (T, cm) change, (**c**) The wet volumetric density (ϱv, g/cm³) and total porosity (ε) change, (d) The reduction of solutions pH after 7 days of membrane incubation.

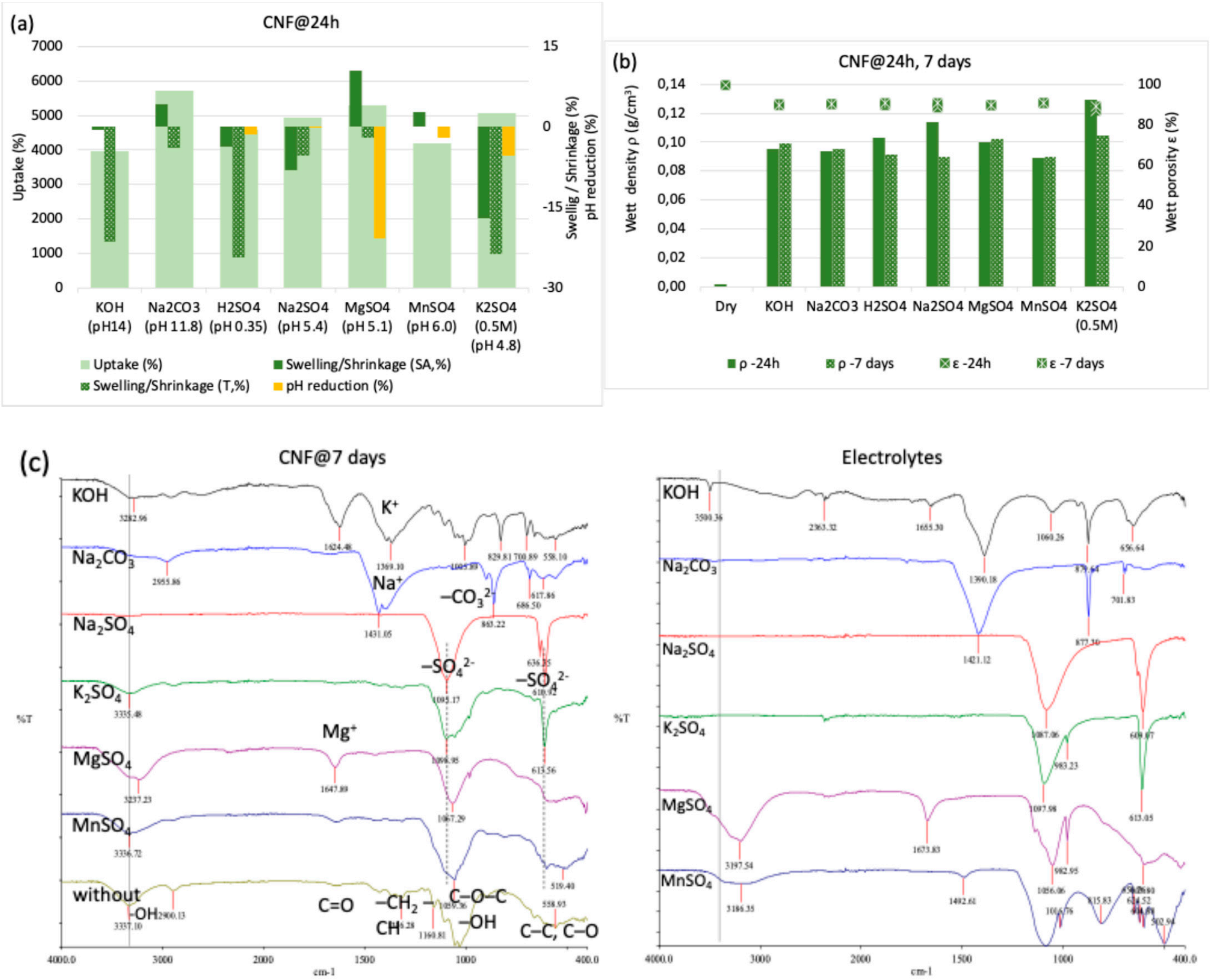

Figure S5. Characterisation of pure CNF-based membranes (without MXene) prepared at -50°C, after 24 h of immersion in different 1/0.5M electrolyte solutions at room temperature: (**a**) The uptake of electrolyte solutions, the swelling/shrinkage percentage based on surface area (SA, ϱ_A, g/cm^2) and thickness (T, cm) change, and pH percentage reduction, (**b**) The wet volumetric density (ϱ_V, g/cm^3) and total porosity (ε) change. (**c**) The FTIR spectra of the CNF-based membranes after 7 days of incubation in electrolytes, compared to the electrolyte powders. Due to different saturation in solubility of electrolytes at room temperature, 1 M vs. 0.5M concentrated solutions have been used. All electrolyte solutions have provided high conductivity that did not change during testing: KOH~157 mS, Na_2CO_3~70 mS, H_2SO_4~336 mS, Na_2SO_4~79 mS, $MgSO_4$~42 mS, $MnSO_4$~36 mS, K_2SO_4~69 mS. The FTIR spectra for the membrane after 7 days of incubation in H_2SO_4 could not be performed due to its complete degradation (Figure S2).

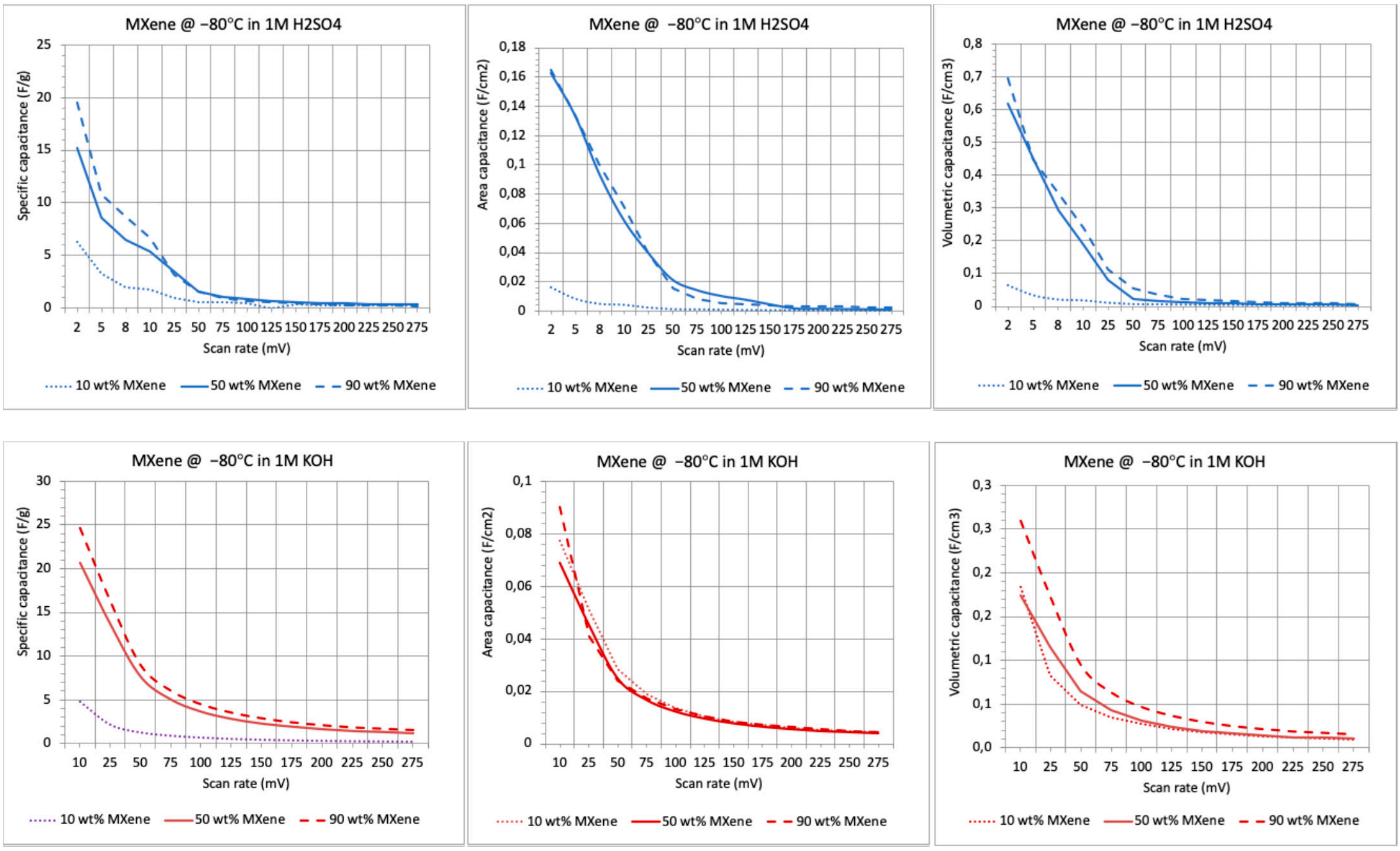

Figure S6. Specific, areal and volumetric capacitances at various scan rates for CNF-based membranes prepared with 10 wt%, 50 wt% and 90 wt% $Ti_3C_2T_x$ MXene (HT-etched) at -80°C freeze-casting temperature, performed in 1M H_2SO_4 and 1M KOH electrolytes.

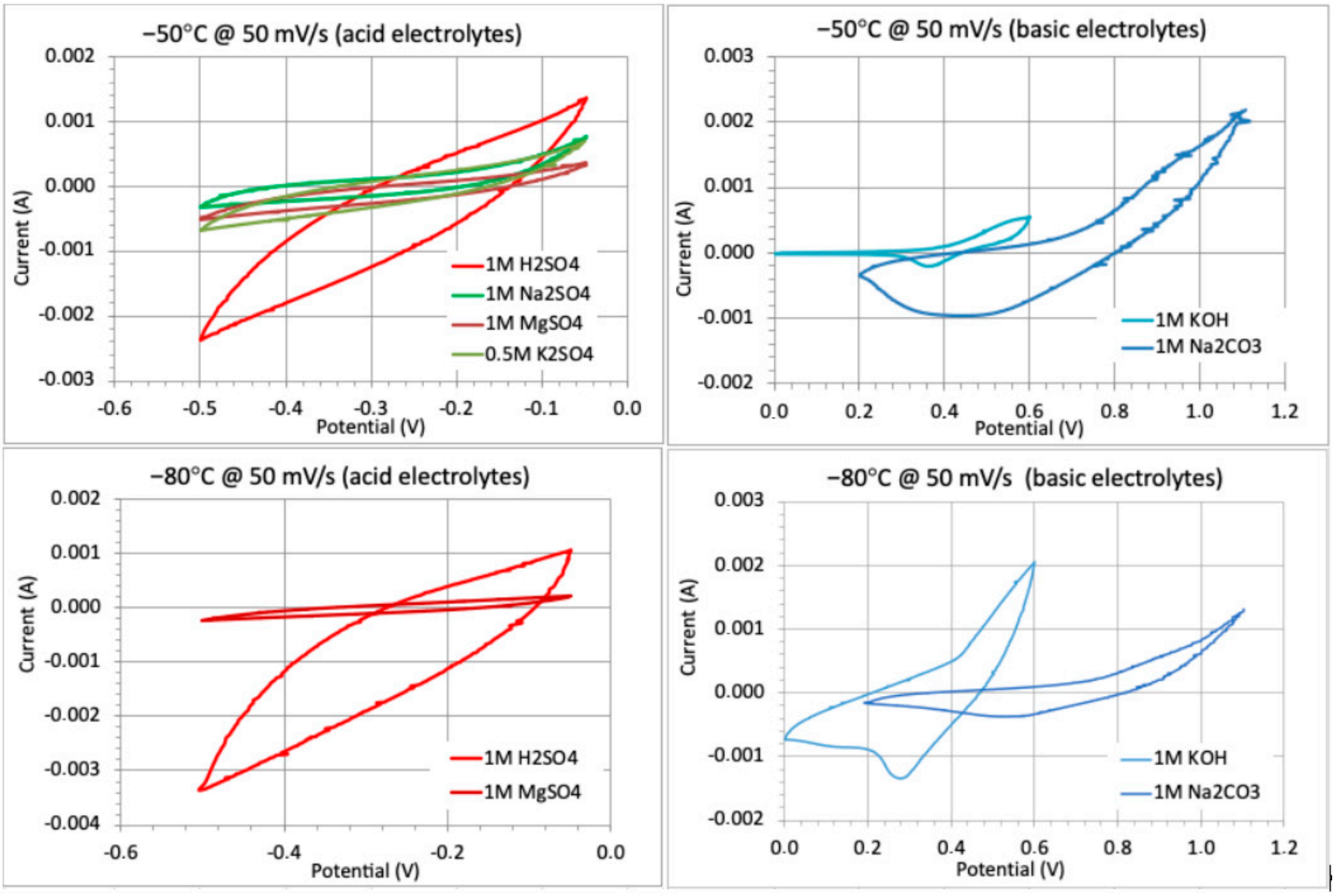

Figure S7. Cyclic voltammogram (CV) curves at scan rates of 50 mV/s for different electrolytes using CNF-based membrane prepared with 50 wt% $Ti_3C_2T_x$ MXene (HT-etched) at -50°C or 80°C.

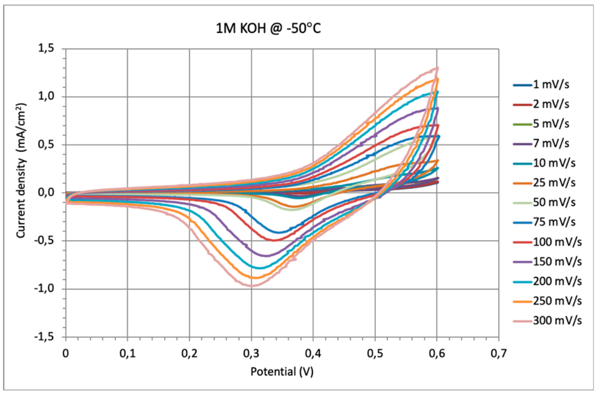
1M KOH @ -50°C
Current density (mA/cm²)
Potential (V)
1 mV/s
2 mV/s
5 mV/s
7 mV/s
10 mV/s
25 mV/s
50 mV/s
75 mV/s
100 mV/s
150 mV/s
200 mV/s
250 mV/s
300 mV/s

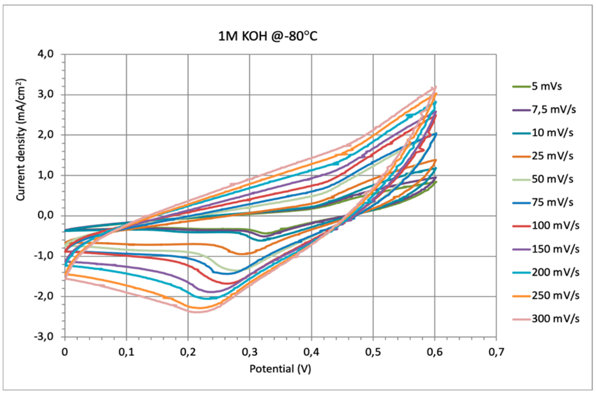
1M KOH @-80°C
Current density (mA/cm²)
Potential (V)
5 mVs
7,5 mV/s
10 mV/s
25 mV/s
50 mV/s
75 mV/s
100 mV/s
150 mV/s
200 mV/s
250 mV/s
300 mV/s

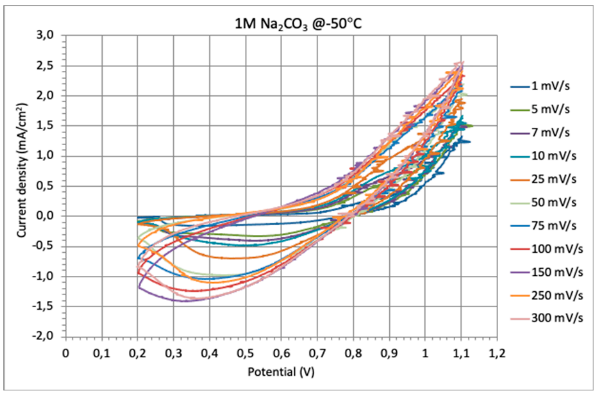
1M Na2CO3 @-50°C
Current density (mA/cm²)
Potential (V)
1 mV/s
5 mV/s
7 mV/s
10 mV/s
25 mV/s
50 mV/s
75 mV/s
100 mV/s
150 mV/s
250 mV/s
300 mV/s

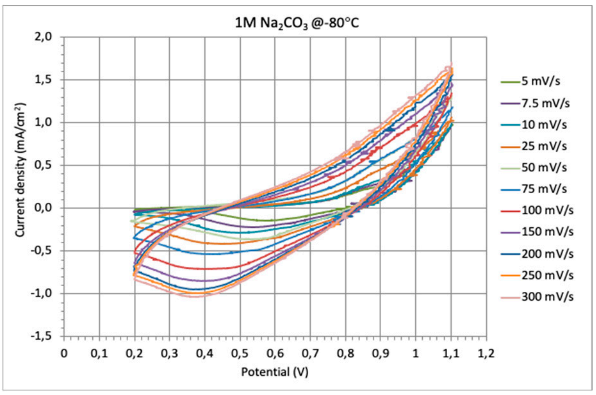
1M Na2CO3 @-80°C
Current density (mA/cm²)
Potential (V)
5 mV/s
7.5 mV/s
10 mV/s
25 mV/s
50 mV/s
75 mV/s
100 mV/s
150 mV/s
200 mV/s
250 mV/s
300 mV/s

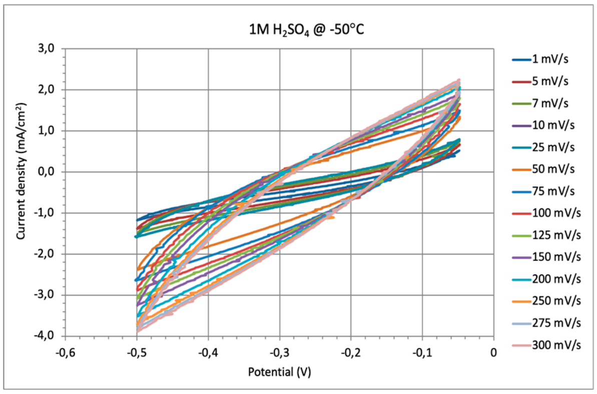
1M H2SO4 @ -50°C
Current density (mA/cm²)
Potential (V)
1 mV/s
5 mV/s
7 mV/s
10 mV/s
25 mV/s
50 mV/s
75 mV/s
100 mV/s
125 mV/s
150 mV/s
200 mV/s
250 mV/s
275 mV/s
300 mV/s

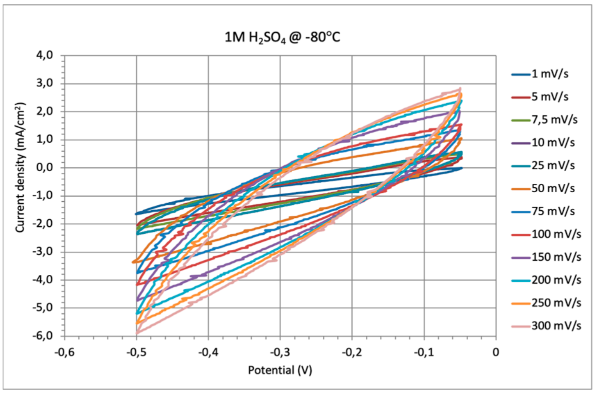
1M H2SO4 @ -80°C
Current density (mA/cm²)
Potential (V)
1 mV/s
5 mV/s
7,5 mV/s
10 mV/s
25 mV/s
50 mV/s
75 mV/s
100 mV/s
150 mV/s
200 mV/s
250 mV/s
300 mV/s

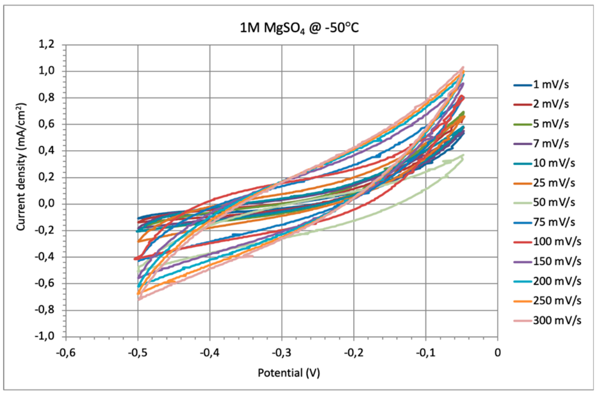
1M MgSO4 @ -50°C
Current density (mA/cm²)
Potential (V)
1 mV/s
2 mV/s
5 mV/s
7 mV/s
10 mV/s
25 mV/s
50 mV/s
75 mV/s
100 mV/s
150 mV/s
200 mV/s
250 mV/s
300 mV/s

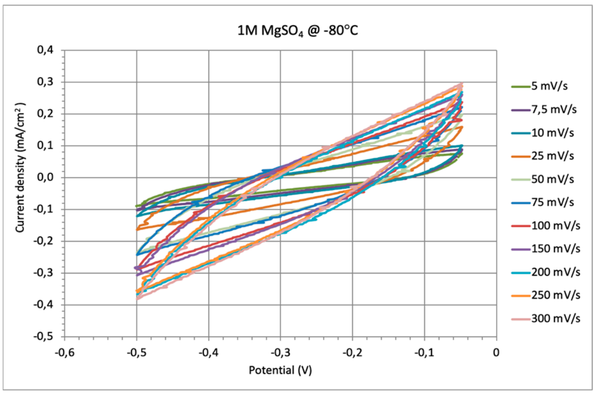
1M MgSO4 @ -80°C
Current density (mA/cm²)
Potential (V)
5 mV/s
7,5 mV/s
10 mV/s
25 mV/s
50 mV/s
75 mV/s
100 mV/s
150 mV/s
200 mV/s
250 mV/s
300 mV/s

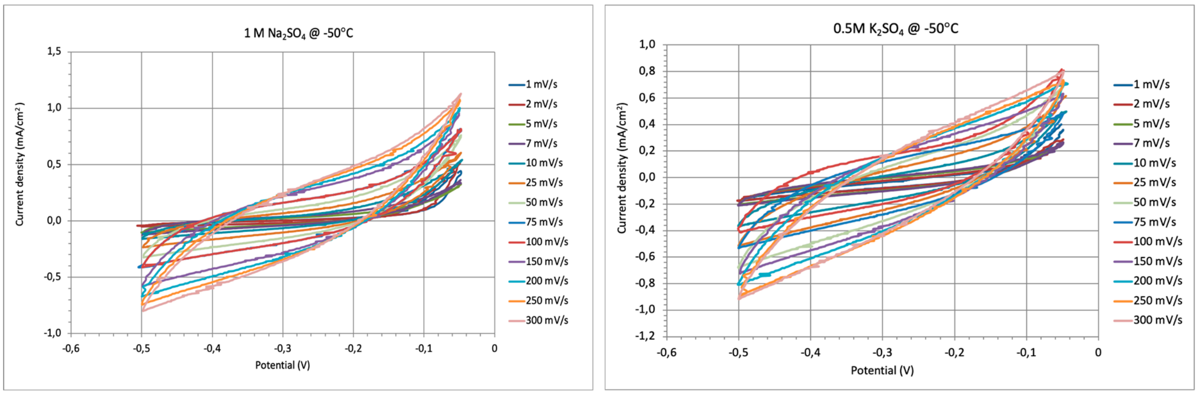

Figure S8. CV curves at various scan rates and different potential windows corresponding to the type of electrolytes for CNF-based membranes prepared with 50 wt% $Ti_3C_2T_x$ MXene (HT-etched) at different freeze-casting temperatures (−50°C vs −80°C).
